# Toxicology of silica nanoparticles: an update

**DOI:** 10.1007/s00204-017-1993-y

**Published:** 2017-06-01

**Authors:** Sivakumar Murugadoss, Dominique Lison, Lode Godderis, Sybille Van Den Brule, Jan Mast, Frederic Brassinne, Noham Sebaihi, Peter H. Hoet

**Affiliations:** 1 Unit for Lung Toxicology, Katholieke Universiteit Leuven, Herestraat 49, O&N1, Room: 07.702, box 706, 3000 Louvain, Belgium; 2 Louvain Centre for Toxicology and Applied Pharmacology (LTAP), Université Catholique de Louvain, Avenue E. Mounier 52/B1.52.12, 1200 Brussels, Belgium; 3 Department of Occupational, Environmental and Insurance Medicine, Katholieke Universiteit Leuven, Kapucijnenvoer 35 block d, box 7001, 3000 Louvain, Belgium; 4 EM-unit, Center for Veterinary and Agrochemical Studies and Research (CODA-CERVA), Groeselenberg 99, Uccle, 1180 Brussels, Belgium; 5 General Quality and Safety, Metrology Department, National Standards, North Gate-Office 2A29, Bd du Roi Albert II, 16, 1000 Brussels, Belgium

**Keywords:** Amorphous silica nanoparticles, Pyrogenic, Colloidal, Stöber, Oxidative stress, Toxicity

## Abstract

Large-scale production and use of amorphous silica nanoparticles (SiNPs) have increased the risk of human exposure to SiNPs, while their health effects remain unclear. In this review, scientific papers from 2010 to 2016 were systematically selected and sorted based on in vitro and in vivo studies: to provide an update on SiNPs toxicity and to address the knowledge gaps indicated in the review of Napierska (Part Fibre Toxicol 7:39, 2010). Toxicity of SiNPs in vitro is size, dose, and cell type dependent. SiNPs synthesized by wet route exhibited noticeably different biological effects compared to thermal route-based SiNPs. Amorphous SiNPs (particularly colloidal and stöber) induced toxicity via mechanisms similar to crystalline silica. In vivo, route of administration and physico-chemical properties of SiNPs influences the toxicokinetics. Adverse effects were mainly observed in acutely exposed animals, while no significant signs of toxicity were noted in chronically dosed animals. The correlation between in vitro and in vivo toxicity remains less well established mainly due to improper—unrealistic—dosing both in vitro and in vivo. In conclusion, notwithstanding the multiple studies published in recent years, unambiguous linking of physico-chemical properties of SiNPs types to toxicity, bioavailability, or human health effects is not yet possible.

## Introduction

Nanosilica, also known as the nanoform (<100 nm) of silicon dioxide or silica nanoparticles (SiNPs), possesses distinct physico-chemical characteristics compared to its bulk form; smaller size materials have an increased surface-to-volume ratio and a higher surface reactivity (Oberdörster 2010; Napierska et al. 2010). Due to their appealing properties, SiNPs are now extensively used in agriculture, food, and consumer products including cosmetics (Napierska et al. 2010; Khot et al. 2012; Kasaai 2015; Brinch et al. 2016). Until 2012, nearly 1.5 million tons of SiNPs had already been placed in the global market (Liljenström et al. 2013) and SiNPs became one of the three most produced nanomaterials (NMs) worldwide in 2013. Among the 846 nano-based products listed in a consumer products inventory, approximately 100 claim to contain SiNPs (Vance et al. 2015). Moreover, amorphous SiNPs are being synthesized with highly tunable biocompatibility and stability, and considered as a very promising candidate for various bio-medical applications such as gene carrier, drug delivery, and molecular imaging (Tang and Cheng 2013; Bitar et al. 2012).

In recent years, large-scale industrial production and global commercialization of SiNPs have resulted in increased risk of human exposures at workplaces (Kim et al. 2014a; Oh et al. 2014). Food additive silica (E551) is also in the nano size range (Dekkers et al. 2011), indicating that the general population is probably more exposed than initially anticipated. Moreover, in view of the efforts to use NM in medical applications, SiNPs could also be intentionally introduced into the human body for disease diagnosis and treatments (Croissant et al. 2017). Such growing potentials for exposure raised a global concern regarding the safety and potential adverse health effects of SiNPs.

Human health effects associated with silica exposure, especially crystalline silica (0.5–10 µm), have widely been studied. Occupational exposure to crystalline silica induces silicosis in workers (a fibrotic lung disease) and is also associated with lung cancer, emphysema, and pulmonary tuberculosis (Leung et al. 2012). Conversely, natural amorphous silica is generally considered as less harmful, since the toxicological potential of silica has so far been linked to its crystallinity. Recent studies have revealed that amorphous SiNPs can be as reactive as crystalline particles (Turci et al. 2016). In vivo, amorphous SiNPs are, however, cleared more rapidly from the lung, which may contribute to explain their lower pathogenic potential (Arts et al. 2007). The human health effects of nanosilica remain to be clarified and toxicologists believe that exposure to SiNPs, due to their small size, may bring different adverse effects compared to micron-sized silica (Napierska et al. 2010).

The comprehensive review of Napierska et al. (2010) suggested that exposure to SiNPs (1–100 nm) induced toxic effects in vitro (immortalized mammalian cell lines) and in vivo (rats and mice). Physico-chemical properties such as size, surface area, and surface features were found to play a key role in the toxicity of SiNPs. Importantly, Napierska concluded that physico-chemical properties of SiNPs differ based on their production method and, therefore, may cause different biological effects. However, no definite conclusions were made due to insufficient or no data available for,

- Detailed physico-chemical characterization of different types of SiNPs;
- Comparison of the toxicity of different types of SiNPs (based on their production process);
- Comparison of the toxicity mechanisms of amorphous SiNPs and crystalline silica;
- Exposure via different routes and adverse effects of chronic exposure in vivo;
- Correlation of in vitro and in vivo studies and
- Physico-chemical properties for the safer design of SiNPs.

**Fig. 1:**
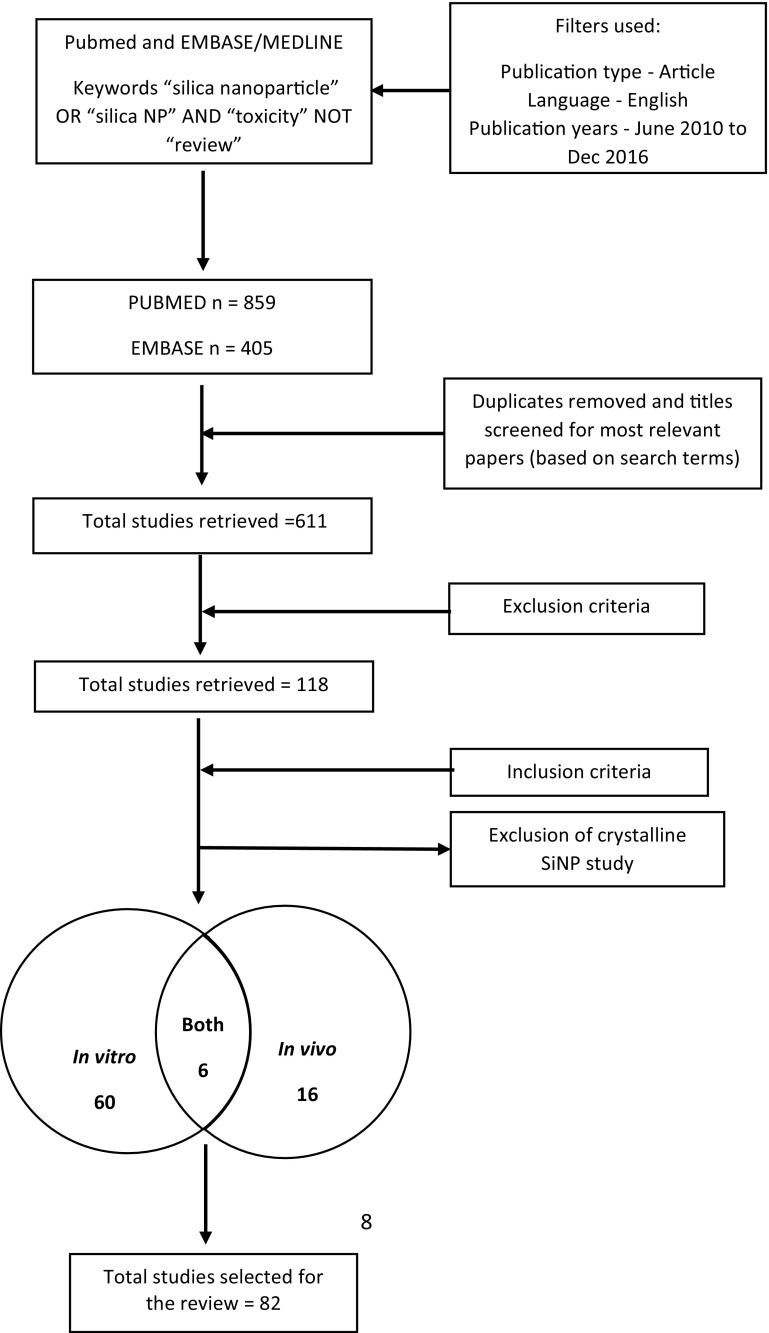
Systematic selection of studies

Therefore, the aim of this review is to summarize the toxicity studies of SiNPs published after the Napierska review (2010), critically discuss the outcomes, and to evaluate how these data gaps have been addressed (Fig. 1).

## Methodology

The selection criteria was similar to the method described in Vriens et al. (2017), which was used to construct the MOD-ENP-TOX nanotoxicity database.

### Exclusion criteria

To specifically focus on adverse health effects of SiNPs, papers reporting on other interventions such as ecotoxicity, synergistic effects, SiNPs doped with other materials, and therapy-based outcomes were excluded.

### Inclusion criteria

Papers reporting,

1. physico-chemical characteristics such as primary size, shape, composition, and crystallinity;
2. toxicological endpoints such as cytotoxicity, apoptosis/necrosis, genotoxicity, oxidative stress, immunotoxicity, and autophagy using immortalized cell lines or primary cells (experimental in vitro studies); and
3. toxic effects in laboratory-animals, more specifically in vivo experiments using rats and mice.

### Literature search

We searched two databases for papers published from June 2010 (after Napierska et al. 2010) to December 2016. In the PubMed “http://www.ncbi.nlm.nih.gov/PubMed” and EMBASE “https://www.embase.com” databases, the following keyword combinations were used: “silica nanoparticle” OR “silica NP” AND “toxicity” NOT “review”. We retrieved a list of 859 and 405 articles in English, respectively. In a second step, duplicates were removed and the titles were screened to identify studies that best matched with our search terms, leaving 611 relevant papers. In a third step, we excluded papers that met the exclusion criteria and left 128 most relevant papers. Finally, 82 papers reporting a minimum set of physico-chemical characterization and toxic effects were selected for the main content of the review. Notably, only one study was found on the toxicity of crystalline nanosilica (Chu et al. 2012), but it was finally excluded due to insufficient data on the size of particles. As a result, the review is dealing only with amorphous SiNPs.

Induction of oxidative stress is considered as the major mechanism involved in SiNPs toxicity (Wang et al. 2009; Ye et al. 2010a, b) and, therefore, the in vitro section of the review was structured according to toxic endpoints and its association with oxidative stress. For in vivo, studies were sorted based on exposure route and modalities, since they can significantly influence the toxicokinetics of SiNPs. Throughout the review, the following abbreviations were used to indicate the different types of silica nanoparticles: SiNPs when it is not clear which type was used, C-SiNPs for colloidal silica; S-SiNP for stöber silica; M-SiNPs for mesoporous silica; Pr-SiNPs for precipitated silica; and Py-SiNPs for pyrogenic silica.

The table summarizing in vitro studies (Table 1) was sorted according to the type of SiNPs (colloidal, stöber, mesoporous, pyrogenic, precipitated, and not specified) and cell types. Table 2 (in vivo studies) was organized according to the type of SiNPs and exposure routes.

**Table 1:** In vitro studies on SiNPs toxicity

Type of SiNPs	Cell line	Cell type	Particle primary size	Source	Exposure dose	Exposure duration	Endpoint	Assay(s)/method(s)	Results	References
C-SiNPs	V79	Hamster lung fibroblast	9,15, 30, and 55 nm	AkzoNobel AB	10–600 µg/ml	24 h	Cell viability	Tryphan blue exclusion and colony formation assay	Reduction with 15 nm NPs	Maser et al. (2015)
	A549	Human type II alveolar epithelial					Genotoxicity	Comet assay	Increase with 15 nm NPs	
C-SiNPs	H441	Human distal lung epithelial	30 nm	Sigma-Aldrich	0.6–600 µg/ml	4–20 h	Cell viability	MTS assay	Reduction at 600 µg/ml in monocultures	Kasper et al. (2011)
	ISO-HAS-1	Human endothelial					Cytotoxicity	LDH assay	Increase at 600 µg/ml in all cultures	
							Cellular barrier integrity	TEER measurement	Reduction at 600 µg/ml in all cultures	
	Co-culture: H441/ISO-HAS-1	Human distal lung epithelial + human endothelial					Pro-inflammatory response(s)	ELISA	Increase of siCAM-1, IL-6 and IL-8	
							Cell morphology	Hoechst staining	Cells rounded at 600 µg/ml	
							Apoptosis	Western blot	Increase of apoptotic proteins	
C-SiNPs	A549	Human type II alveolar epithelial	70 nm—with or without lung surfactant	Micromod Partikeltechnologie	100 µg/ml	4–20 h	Cell viability	MTS and crystal violet assay	Reduction in monocultures	Kasper et al. (2015)
							Cytotoxicity	LDH assay	Increase in monocultures	
							Pro-inflammatory response(s)	ELISA	Increase of IL-8 in monocultures and co-cultures	
	Co-culture: A549 and ISO-HAS-1	Human type II alveolar epithelial + human endothelial					Cellular uptake	Hoechst staining	Increase in monocultures and co-cultures	
C-SiNPs	Caco2	Human colon epithelial	15 nm (Levasil 200/40%) and 55 nm (Levasil 50/50%)	H.C. Starck	0.03–156.3 µg/cm^2^	24, 48 and 72 h	Cell viability	XTT assay	Dose-dependent reduction with 15 nm NPs	Tarantini et al. (2015a, b)
							Oxidative stress	DCFH-DA assay	Dose-dependent increase with 15 nm SiNPs	
							Pro-inflammatory response(s)	ELISA	Increase of IL-8 with 15 nm NPs only at 156.3 µg/cm^2^	
							Apoptosis	Caspase-3 assay	Dose-dependent increase with 15 nm NPs	
							Genotoxicity	Micronuclei induction	Dose-dependent increase with 15 nm NPs	
							Genotoxicity	ɣH2AX fluorescence	Increase with 15 nm NPs	
							NP internalization	TEM	NPs detected in lysosomes and in endocytic compartments	
C-SiNPs	HepG2	Human liver epithelial	19, 43, 68, and 498 nm	Laboratory synthesis	12–200 µg/ml	24 h	Cell viability	CCK-8 assay	Size and dose-dependent reduction	Li et al. (2011)
							Cytotoxicity	LDH assay	Size and dose-dependent increase	
							Oxidative stress	DCFH-DA assay	Size-dependent increase	
							Genotoxicity	Comet assay	Size-dependent increase	
							Apoptosis	Apoptosis assay	Size-dependent increase	
							Cell cycle arrest	Flow cytometry	Size-dependent increase	
							Cell morphology	H & E staining	Cellular shrinkage, chromatin condensation and vacuolar degeneration detected	
C-SiNPs	RAW 264.7	Mouse blood macrophage	20 and 100 nm (uncoated or l-arginine coated)	E&B Nanotech	10–640 µg/ml	24 h	Cell viability	WST-8 assay	Size and surface charge-dependent reduction	Kim et al. (2014b)
C-SiNPs and M-SiNPs	J774A.1	Mouse macrophage	100 nm	Laboratory synthesis	0.1–1000 µg/ml	24 and 72 h	Cell viability	MTT assay	Reduction only for C-SiNPs SiNPs	Lee et al. (2011)
							Apoptosis	Annexin V/PI staining	Increase of caspase-3 activation	
							Pro-inflammatory response(s)	RT PCR and western blot	Increase of TNF-α, IL-6 and IL-1β	
							Pathway analysis	RT PCR and western blot	Activation of MAPKs and NF-kB	
C-SiNPs	J744A.1	Mouse macrophage	25, 46, 183,182, and 188 nm	Sigma-Aldrich	–	24 h	Cell viability	WST-1 assay	ED50: 6–9 µg/ml and 15–22 µg/ml in J774 and 3T3, ,respectively	Rabolli et al. (2011)
	BALB/c3T3	Mouse fibroblast					In vitro dosimetry	ISDD simulation	Similar delivered doses for all particle sizes	
C-SiNPs	L5178Y/Tk^+/−^	Mouse lymphoma	7 nm	Sigma-Aldrich	0.01–150 µg/ml	4 h	Genotoxicity	Lymphoma assay	Mutations detected at 100 and 150 µg/ml	Demir and Castranova (2016)
C-SiNPs	PBMCs	Lymphocytes, monocytes and dendritic cells	10 and 100 nm	Polysciences	50–2000 µg/ml	24 and 48 h	Cell viability	FACS	Dose, time and size-dependent reduction	Mendoza et al. (2014)
							Oxidative stress	GSH depletion	Size and dose-dependent increase	
							Oxidative stress	Western-blot	Dose-dependent increase in proteins with free radicals only with 10 nm	
							Pro-inflammatory response(s)	Multiplex bead array	Size and dose-dependent increase in cytokines	
							Cell morphology	Immuno electron microscopy	Size-dependent increase in cell damage	
C-SiNPs	HUVEC	Human vein endothelial	10, 50, 150, and 500 nm	Polysciences	10 nm–10 μg/ml 50, 150 nm and 500 nm–50 μg/mL	1 h (10 nm) and 3 h (50–500 nm)	Oxidative stress	Fluorescent microscopy	Free radical increase	Corbalan et al. (2011)
							Pro-inflammatory response(s)	Cytometric bead array	Size-dependent increase of IL-6 and IL-8	
							Oxidative stress	Nanosensors	Imbalance in [NO]/[ONOO−]	
							Inflammatory factors	RT-PCR	Size-dependent increase of ICAM1, VCAM1, SELE, MMP9, COX2 and F3	
							NF-κB-binding activity	ELISA	Increase of NF-κB-binding activity	
							NP internalization	TEM	NPs detected in cytoplasm and vesicles	
C-SiNPs	Platelets	Mouse	50 nm	Polysciences	1, 5 and 25 µg/ml	30 min	Platelet aggregation	Aggregation assay	Dose-dependent increase	Nemmar et al. (2015)
							Cytotoxicity	LDH assay	Dose-dependent increase	
							Oxidative stress	LPO assay	Dose-dependent increase of MDA formation	
							Calcium concentration	Fluorimeter	Increase in calcium concentration at 25 µg/ml	
C-SiNPs	Platelets	Human	10, 50, 150, and 500 nm	Polysciences	1–200 μg/ml	15 min	Platelet aggregation	Aggregation assay	Size-dependent increase	Jose Corbalan et al. (2012)
							Oxidative stress	Nanosensors	Size-dependent increase of NO	
							SELP and GPIIb/IIIa expression	Flow cytometry	Increase with 10, 100 and 150 nm	
							Morphology	TEM	Platelets strongly aggregated	
S-SiNPs	A549	Human type II alveolar epithelial	2.1, 16.4, 60.4, and 104 nm	Laboratory synthesis	5 µg/cm^2^ of plate surface	12–24 h	Cell viability	MTT assay	Size-dependent reduction	Napierska et al. (2012b)
	THP-1	Human monocyte					Cytotoxicity	LDH assay	Size-dependent increase	
	Biculture: A549/THP-1	Lung co-cultures					Pro-inflammatory response(s)	Cytometric bead array	Increase of all cytokines in the presence of 2 nm and 60 nm (except TNF-α)	
	Triculture: A549/THP-1/EA.hy926	Lung co-cultures								
S-SiNPs	Co-culture: NCI-H441/ISO-HAS-1 with or without THP-1 cells	Lung co-cultures	15, 35, and 80 nm	Laboratory synthesis	50 and 100 µg/ml	72 h	Oxidative stress	DCFH-DA assay	Increase in the presence of THP-1	Farcal et al. (2012)
							Pro-inflammatory response(s)	ELISA	Increase of IL-8 and TNF-α	
							Surfactant protein expression	RT-PCR	Increase of surfactant proteins in the presence of THP-1	
							Cellular barrier integrity	TEER measurement	No effect	
S-SiNPs	HepG2	Human liver epithelial	43 nm	Laboratory synthesis	25–200 µg/ml	3 and 24 h	Oxidative stress	DCFH-DA assay	Dose-dependent increase	Sun et al. (2011)
							Mitochondrial membrane potential	Probe measurements	Dose-dependent increase	
							Apoptosis	Annexin V/PI staining and western blot	Dose-dependent increase	
							Cell morphology	TEM	NPs detected in cytoplasm, mitochondria and lysosomes	
S-SiNPs	HepG2	Human liver epithelial	62 nm	Laboratory synthesis	25–100 µg/ml	24 h	Cell viability	MTT assay	Dose-dependent reduction	Yu et al. (2014)
							Oxidative stress	DCFH-DA assay	Dose-dependent increase	
							Autophagy	MDC staining	Dose-dependent increase	
							Autophagy	Immunoblot	Dose-dependent increase of LC3-II/LC3-I	
							NP internalization	LSCM	NPs detected in cytoplasm and mitochondria	
S-SiNPs	LC-02	Human liver epithelial	50 nm	Laboratory synthesis	50–200 µg/ml	24 h	Cell viability	CCK-8 assay	Dose-dependent reduction	Wang et al. (2013)
							Oxidative stress	DCFH-DA assay	Dose-dependent increase	
							Apoptosis	Annexin V/PI staining	Dose-dependent increase	
							Mitochondrial damage	Mitotracker/laser confocal microscopy	Dose-dependent increase	
							Cell morphology	Hoechst staining	Dose-dependent increase in cell damage	
S-SiNPs	HaCaT	Human keratinocyte	50 nm	Laboratory synthesis	25–500 µg/ml	4 h	Cell viability	MTT assay	Dose-dependent reduction	Liang et al. (2014)
							Cytotoxicity	LDH assay	Dose-dependent increase	
							Oxidative stress	DCFH-DA assay	Dose-dependent increase	
							Oxidative stress	GSH depletion	Increase	
							Apoptosis	Hoechst/PI staining	Increase	
							NP internalization	TEM	Detected in cytoplasm	
S-SiNPs	EA.hy926	Human endothelial	16 (pure or iron-doped) and 60 nm	Laboratory synthesis	25 and 50 µg/ml	24 h	Cell viability	MTT assay	Reduction at 50 µg/ml with 16 nm NPs	Napierska et al. (2012a)
							Cytotoxicity	LDH assay	Increase at 50 µg/ml with 16 nm NPs	
							Oxidative stress	DCFH-DA assay	Dose-dependent increase only with Fe doped NPs	
							Oxidative stress	GSH depletion	Dose-dependent increase only with Fe doped NPs	
							Oxidative stress	LPO assay	Dose-dependent increase of MDA formation with Fe doped NPs	
							Oxidative stress	RT-PCR	Increase with 16 nm NPs	
							NP internalization	TEM	NPs found in cytoplasm	
S-SiNPs	HUVEC	Human vein endothelial	62 nm	Laboratory synthesis	25–100 µg/ml	6, 12 and 24 h	Cell viability	MTT assay	Dose-dependent reduction	Duan et al. (2013a)
							Cytotoxicity	LDH assay	Dose-dependent increase	
							Oxidative stress	DCFH-DA assay	Dose-dependent increase	
							Oxidative stress	LPO assay	Dose-dependent increase of MDA formation	
							Oxidative stress	SOD assay	Dose-dependent decrease	
							Oxidative stress	GSH Px assay	Dose-dependent decrease	
							Apoptosis	Annexin V/PI staining	Dose-dependent increase	
							Mitochondrial membrane potential	Probe measurements	Dose-dependent increase	
							Genotoxicity	Comet assay	Dose-dependent increase	
							Cell cycle analysis	Western blot	Dose-dependent upregulation of chk 1 and down regulation Cdc25c, Cyclin B1, Cdc2	
							NP internalization	LSCM and TEM	NPs detected in cytoplasm	
S-SiNPs	HUVEC	Human vein endothelial	62 nm	Laboratory synthesis	25–100 µg/ml	24 h	Autophagy	MDC staining	Dose-dependent increase of LC-3 II/LC3-I	Duan et al. (2013b)
							Autophagy	LSCM and TEM	Dose-dependent increase in cellular uptake and autophagic vacuoles, autophagosomes and autolysosomes detected	
							Expression of inflammatory factors	ELISA	Dose-dependent increase in the expression of CRP, TNF-α, IL-1β and IL-6	
							Oxidative stress	NO, NOS, eNOS and iNOS measurements	Dose-dependent increase of iNOS and decrease of NO, NOS and eNOS	
							Pathway analysis	Western blot	Dose-dependent decrease of p-mTOR/mTOR, p-P13 K/P13 K and p- Akt/Akt	
S-SiNPs	HUVEC	Human vein endothelial	58 nm	Laboratory synthesis	12.5–100 µg/ml	24 h	Cell viability	MTT assay	Dose-dependent reduction	Guo et al. (2015)
							Cytotoxicity	LDH assay	Dose-dependent increase	
							Oxidative stress	DCFH-DA assay	Dose-dependent increase	
							Oxidative stress	GSH depletion	Dose-dependent increase	
							Oxidative stress	LPO assay	Dose-dependent increase of MDA formation	
							Oxidative stress	SOD assay	Dose-dependent decrease	
							Oxidative stress	GSH-Px assay	Dose-dependent decrease	
							Inflammatory factors	ELISA	Increase of IL-1β, IL-8, TNFα, ICAM-1, VCAM-1, and MCP-1	
							Oxidative stress	NO, NOS, eNOS and iNOS measurements	Differential expression of NO, iNOS and eNOS activity and downregulation of ET -1	
							Pathway analysis	RT-PCR and western blot	Dose-dependent increase of Nrf-2, p-ERK, p-JNK, p-p38 MAPK and NF-kB	
S-SiNPs	HUVEC	Human vein endothelial	62 nm	Laboratory synthesis	25–100 µg/ml	24 h	Cellular uptake	LSCM	Dose-dependent increase	Duan et al. (2014b)
							Cytoskeleton damage	Cell cytoskeleton staining	Weakening of F actin at 100 µg/ml	
							Mitochondrial membrane potential	Probe measurements	Dose-dependent increase	
							Autophagy	TEM	Autophagic ultrastructures detected	
							Autophagy	Western blot	Increase of LC3-II/LC3-I	
							Inflammatory factors	Western blot	Decrease of ICAM-1 and VCAM-1	
							Pathway analysis	Western blot	Dose-dependent decrease of p-mTOR/mTOR, p-P13K/P13K and p-Akt/Akt	
S-SiNPs	HUVEC	Human vein endothelial	58 nm	Laboratory synthesis	12.5–100 µg/ml	24 h	Cell viability	MTT assay	Size and dose-dependent reduction	Guo et al. (2016)
							Cytotoxicity	LDH assay	Size and dose-dependent increase	
							Oxidative stress	DCFH-DA assay	Increase	
							Oxidative stress	LPO, SOD and GSH assay	Increase	
							Redox relative factors	RT-PCR	Increase in Nrf2 activation	
							Apoptosis	AO/EB staining	Increase	
							Mitochondrial membrane potential	Probe measurements	Decrease	
							Autophagy	TEM	Autophagosomes and autophagic vacuoles detected	
							Cellular uptake	ICP-AES	Increase	
							Pathway analysis	Western blot	Increase of MAPK/Bcl-2 and PI3 K/Akt/mTOR signaling	
S-SiNPs and M-SiNPs	MPMCs	Murine peritoneal mast cells	25 nm	Laboratory synthesis	100 µg/ml	24 h	Cell viability	MTT assay	Reduction with non-porous NPs	Maurer-jones et al. (2010)
	RBCs	Human red blood cells					Hemolysis of RBCs	Hemolysis assay	Increase with non-porous NPs	
	Co-culture: MPMC/3T3	Lung co-cultures					Cellular uptake	TEM and ICP-AES	M-SiNPs NPs internalized more than non-porous	
S-SiNPs and M-SiNPs	A549	Human type II alveolar epithelial	115 nm (with or without amine modification)	Laboratory synthesis	10, 50, 100, 250 and 500 μg/ml	24 h	Cell viability	WST-8 assay	Dose-dependent reduction only in RAW 264.7. Amine modified SiNPs were less toxic	Yu et al. (2011)
	RBCs	Human red blood cells								
	RAW 264.7	Mouse macrophage					Hemolysis of RBCs	Hemolysis assay	Increase only with M-SiNPs	
							Cytotoxicity	PI staining	Increase with M-SiNPs	
							Cellular uptake	ICP-MS	S-SiNPs internalized more than M-SiNPs	
M-SiNPs	HT-29	Human colon epithelial	25 and 100 nm	Laboratory synthesis	10–150 µg/ml	24 h	Cell viability	SRB	No reduction	Sergent et al. (2012)
							Cell viability	Impedancemetry	No reduction with 25 nm NPs; higher toxicity at lower doses with 100 nm NPs	
							Cell viability	Flow cytometry	Limited toxicity with 25 nm; higher toxicity at lower doses with 100 nm NPs	
							Genotoxicity	ɣH2AX fluorescence	No effect with 25 nm; greater DNA damage at lower doses with 100 nm NPs	
M-SiNPs	NRK-52E	Rat kidney epithelial	198 nm	Laboratory synthesis	25–1000 µg/ml	3 and 24 h	Cell viability	MTT assay	Dose-dependent reduction	Chen et al. (2015)
							Cytotoxicity	LDH assay	Dose-dependent increase	
							Expression of inflammatory factors	Western blot	Dose-dependent increase of FN, TGF-β, and ICAM-1 (50–400 μg/ml)	
							Pathway analysis	Immuno-fluorescence staining	Dose-dependent increase in the expression of NF-κB p65 (50–400 μg/ml)	
M-SiNPs	HEK293	Human kidney epithelial	100 nm, 2.3 nm pore size	Laboratory synthesis	100 µg/ml	24, 48 and 72 h	Cellular morphology	TEM	Cells shrunk and nucleus condensed	Zhang et al. (2015)
							Oxidative stress	Fluorescent dihydroethi-dium	No effect	
							Oxidative stress	RT PCR	No effect	
							Chromosomal aberrations	FISH assay	No effect	
							Mutations	EGFR and KRAS	No effect	
							Genotoxicity	Human mRNA micro array	579 genes upregulated and 1263 genes downregulated	
M-SiNPs	EA.hy926	Human endothelial	48 nm (surface functionalized with PET and TMS)	Laboratory synthesis	20–1000 µg/ml at a flow rate of 30 µl/min using a microfluidic device	–	Cell viability	Tryphan blue	No effect	Kim et al. (2014a)
	Platelets (activated and non-activated)	Human					Platelet adhesion	Adhesion assay	Increase at 1000 µg/ml	
							Platelet aggregation	Aggregation assay	Increase at 1000 µg/ml	
							Morphology	Fluorescent fixed cell imaging	Large aggregate formation at 1000 µg/ml	
Py-SiNPs and S-SiNPs	A549	Human type II alveolar epithelial	12 (Py-SiNPs) and 50 nm(S-SiNPs)	Py-SiNPs—Evonik; S-SiNPs—Landsberg am Lech	52 μg/cm^2^ and 117 μg/cm^2^ (ALI) 15.6 μg/cm^2^ (sub-merged)	ALI (5 and 7 h) Sub-merged (24 h)	Cytotoxicity	LDH assay	Dose-dependent increase	Panas et al. (2014)
							Pro-inflammatory response	ELISA	Increase in IL-8	
							Expression of inflammatory factors	Western blot	Increase in the expression of COX-2 and phosphorylated p38; stronger effects observed in sub-merged culture	
Pr-SiNPs	A549	Human type II alveolar epithelial	15 nm	Nanoamor	25–200 µg/ml	72 h	Cell viability	MTT assay	Dose-dependent reduction	Ahamed (2013)
	A431	Human skin epithelial					Cytotoxicity	LDH assay	Dose-dependent increase	
							Oxidative stress	DCFH-DA assay	Dose-dependent increase	
							Oxidative stress	LPO assay	Dose-dependent increase of MDA formation	
							Oxidative stress	GSH depletion	Dose-dependent increase	
							Apoptosis	RT PCR	Dose-dependent increase of caspase 3 and caspase 9	
Pr-SiNPs (NM-200) and Py-SiNPs (NM-203); NRT-808, NRT-817, NRT-820 and NRT-944	Immortalized balb/3T3	Mouse fibroblast	10–25, 5–30, 15,35, 80, and 90 nm	NM-JRC repository; NRT-Laboratory synthesis	1–100 µg/ml	72 h	Cell viability	MTT assay	Reduction with 15 nm NPs	Uboldi et al. (2012)
							Cell viability	Colony formation assay	No effect	
							Cell viability	Cell transformation assay	No effect	
							Genotoxicity	Micronuclei induction	No effect	
							NP internalization	Fluorescent microscopy	NPs detected in cytoplasm	
Two Py-SiNPs samples (different sizes) one Pr-SiNPs and two C-SiNPs (different sizes)	V79	Hamster lung fibroblast	20–80 nm	SiNPs	12.5–100 µg/cm^2^	24 h	Cell viability	WST-1 assay	Dose-dependent reduction	Guichard et al. (2016)
							Oxidative stress	DCFH-DA assay	No effect	
							Apoptosis	Caspase-3 assay	Dose-dependent increase	
							Genotoxicity	FPG modified comet assay	Dose-dependent increase	
							Genotoxicity	Micronuclei induction	No effect	
							Mutation	HPRT test	No effect	
Py-SiNPs(12 and 40 nm) and S-SiNPs (200 nm)	HT-29	Human colon epithelial	12, 40, and 200 nm	Py-SiNPs—Evonik; S-SiNPs—Angström Sphere	0.03–156.3 µg/cm^2^	24 h	Cell viability	WST-1 assay	Size-dependent reduction	Gehrke et al. (2013)
							Cell viability	SRB assay	Size-dependent reduction	
							Cytotoxicity	LDH assay	Size-dependent increase	
							Oxidative stress	DCFH-DA assay	No effect	
							Oxidative stress	GSH depletion	Dose-dependent increase with 12 nm NPs	
							Pathway analysis	Western blot	Dose-dependent increase of ERK1/2 phosphorylation with 12 nm	
Py-SiNPs	C2BBe1	Human colon epithelial	12 nm	Sigma-Aldrich	10 µg/cm^2^	Acute—24 h Chronic—29 passages	Cell viability	MTT assay	No effect	McCracken et al. (2013)
							Cytotoxicity	LDH assay	No effect	
							Apoptosis	Annexin V/PI staining	No effect	
							Necrosis	Sytox red staining	No effect	
							Cellular uptake	TEM	Uptake only by a fraction of cells	
Py-SiNPs and Pr-SiNPs	GES-1	Human gastric epithelial	10–50 nm	Evonik and Haihua	10–600 µg/ml	24, 48 and 72 h	Cell viability	CCK-8 assay	Size and dose-dependent reduction	Yang et al. (2014b)
	Caco2	Human colon epithelial					Cytotoxicity	LDH assay	Size and dose-dependent increase	
							Oxidative stress	DCFH-DA assay	No effect	
							Apoptosis	Apoptosis assay	No effect	
							Cell cycle arrest	Cell cycle assay	Increase at S phase for GES cells and G2/M for caco2 cells	
							Cellular uptake	TEM	NPs detected in cytoplasm	
							Cellular morphology	Ultramicrotome and TEM	Increase in cell damage	
							Cellular barrier integrity	TEER measurement	No effect	
Py-SiNPs (NM 203) and Pr-SiNPs (NM 202)	MH-S and RAW 264.7	Mouse macrophages	14 nm	JRC repository	5 or 10 µg/cm^2^	24 h	Cytotoxicity	HCSA	Py-SiNPs induced greater reduction than Py-SiNPs	Di Cristo et al. (2016)
							Cellular uptake	He Ion microscopy and flow cytometry	Higher for Py-SiNPs	
							Macrophage activation	RT-PCR	Increase	
							Pro-inflammatory cytokines	ELISA	Increase of TNF-α, IL-6 and IL-1β	
							Oxidative stress	Fluorescence microscopy	No ROS induction	
Pr-SiNPs	HePG2	Human liver epithelial	14 nm	Nanoamor	1–200 µg/ml	72 h	Cell viability	MTT and NRU assay	Dose-dependent reduction at 25–200 µg/ml	Ahmad et al. (2012)
							Oxidative stress	DCFH-DA assay	Dose-dependent increase	
							Oxidative stress	LPO assay	Dose-dependent increase MDA formation	
							Oxidative stress	GSH depletion	Dose-dependent increase	
							Apoptosis	RT PCR and western blot	Up regulation of p53, bax, and caspase 3 and down regulation of bcl 2	
							Apoptosis	Caspase-3 assay	Dose-dependent increase	
Pr-SiNPs (NM 200 & 201), Py-SiNPs (NM 202 & 203)	Human peripheral lymphocytes	Human	14–16 nm	JRC repository	200–1250 µg/ml	24 h	Genotoxicity	Cytokinesis block micronuclei induction	No effect	Tavares et al. (2014)
S-SiNPs	Primary Microglial cells	Rat macrophage like cells	150–200 nm	Laboratory synthesis	0.0728–7.28 µg/ml	24 h	Cell viability	MTS assay	No effect	Choi et al. (2010)
							Cytotoxicity	LDH assay	No effect	
							Oxidative stress	DCFH-DA assay	No effect	
							NO production	DAF-FM assay	No effect	
							Pro-inflammatory response(s)	Luminex	Reduction in TNF-α. A small amount of IL-β detected	
							Inflammatory factors	RT-PCR	Increase in COX-2	
Reverse microemulsion	HK-2	Human kidney epithelial	20 and 100 nm	Laboratory synthesis	5–500 µg/ml	24,48 and 72 h	Cell viability	WST-1 and clonogenic assay	Size, dose and time-dependent reduction	Passagne et al. (2012)
	LLC PK-1	Porcine kidney epithelial					Oxidative stress	LPO assay	Dose-dependent increase of MDA formation	
							Oxidative stress	Fluorescent dihydroethi-dium	Dose-dependent increase	
							Oxidative stress	RT-PCR	Upregulation of anti-oxidant genes	
							Cellular uptake	TEM	Internalization of particles	
SiNPs	A549	Human type II alveolar epithelial	20 nm (with or without amine or carboxyl coated)	Laboratory synthesis	200–1000 µg/ml	12–48 h	Cell viability	PI staining	Reduction above 250 µg/ml	Nowak et al. (2014)
							Autophagy	MDC staining	Threefold and fivefold increase at 100 and 1000 µg/ml ,respectively	
							Autophagy	RT-PCR	ATG-12 and BECN genes upregulated at 1000 µg/ml	
							Cellular uptake	Immunostaining and fluorescent microscopy	Functionalized particles efficiently taken up than bare SiNPs	
							Cellular morphology	TEM	Cell blebbing detected	
SiNPs	BEAS-2B	Human bronchial epithelial	20–40 nm	Sigma-Aldrich	1 µg/ml	24 h	Cell viability	MTT assay, flow cytometry	Reduction in cell viability	Eom and Choi (2011)
							Oxidative stress	DCFH-DA assay	Increase in ROS production	
							Pathway analysis	Western blot	Increase in HO-1, Nrf-2 and ERK phosphorylation	
SiNPs	HFL-1	Human lung fibroblast	20 and 80 nm	Laboratory synthesis	250–2000 µg/ml	48 h	Cell viability	MTT assay	Size and dose-dependent reduction	Xu et al. (2012)
							Oxidative stress	DCFH-DA assay	Size and dose-dependent increase	
							Apoptosis	Flow cytometry	Size and dose-dependent increase	
							Apoptotic pathway analysis	Western blot	Increase of p53 and differential expression of cytochrome C, Bax, Bcl‐2, caspase, β‐actin and COX IV	
SiNPs	MRC-5	Human lung fibroblast	4–13 nm	NaBond Technologies	12.5–62.5 µg/ml for 24, 48 and 72 h (cell viability) Other assays—62.5 µg/ml	Cell viability—48 and 72 h Other assays—24, 48 and 72 h	Cell viability	MTT assay	Time and dose-dependent reduction	Voicu et al. (2015)
							Oxidative stress	DCFH-DA assay	Time-dependent increase	
							Oxidative stress	GSH depletion	Time-dependent increase	
							Advanced oxidation protein products	Western blot	Time-dependent increase	
							Apoptosis	PI staining	Time-dependent increase	
							Autophagy	MDC staining	Time-dependent increase of LC-3 II/LC3-I	
							Cellular morphology	H & E staining	Vacuolization of cytoplasm detected	
SiNPs	A549	Human type II alveolar epithelial	30 nm	Nanoamor	0.0–100 µg/ml	24 h	Cell viability	MTT assay	Dose-dependent reduction in A549	Michael Berg et al. (2013)
	MeT-5A	Human bronchial epithelial					Oxidative stress	DCFH-DA assay	Dose-dependent increase in A549	
							Oxidative stress	GSH depletion	Dose-dependent increase in A549	
							Oxidative stress	Western blot and RT PCR	Differential expression of anti-oxidant proteins	
							Oxidative stress	Nrf 2 Immunofluorescence	Increase of Nrf 2 in A549	
							Oxidative stress	Catalase assay	Dose-dependent increase	
SiNPs	HFL-1	Human lung fibroblast	20 nm	Nanjing High Technology of Nano	250–1500 µg/ml	48 h	Cell viability	MTT assay	Dose-dependent reduction	Zhang et al. (2011)
							Apoptosis	Fluorescence microscopy	Dose-dependent increase	
							Cellular morphology	Phase contrast microscopy	Detection of morphological changes and NPs accumulation	
SiNPs	HaCaT	Human keratinocyte	15, 30, 100, and 5000 nm	Wan Jing New Material Co. Ltd and Sigma-Aldrich (5000 nm)	0.5–100 µg/ml	24 h	Cell viability	CCK-8 assay	Size and dose-dependent reduction	Gong et al. (2012)
							Oxidative stress	DCFH-DA assay	Size and dose-dependent increase	
							Apoptosis	Annexin V/PI staining	Size and dose-dependent increase	
							Genotoxicity	8-OH-dG	Size and dose-dependent increase	
							Genotoxicity	ɣH2AX fluorescence	Size and dose-dependent increase	
							Genotoxicity	Comet assay	Size and dose-dependent increase	
							Cellular morphology	Microscopy	Morphology affected at 10 µg/ml	
SiNPs	HaCaT	Human keratinocyte	70, 300, and 1000 nm	Micromod Partikeltechnologie	10–1250 µg/ml	24 h	Cytotoxicity	LDH assay	Dose-dependent increase	Nabeshi et al. (2011a)
							Oxidative stress	DCFH-DA assay	Dose-dependent increase	
							Oxidative stress	Hydroxyl fluorescein assay	Dose-dependent increase	
							Genotoxicity	8-OH-dG	Increase in tail length and tail moment	
							Cellular uptake	Assessment using Cytochalasin D and apocynin	Internalization of NPs by NADPH oxidase independent endocytosis	
SiNPs	HaCaT	Human keratinocyte	70, 300, and 1000 nm	Micromod Partikeltechnologie	100 µg/ml	24 h	NP internalization	TEM	70 nm NPs into the nucleus and 300 and 1000 nm SiNPs only in endosomes	Nabeshi et al. (2011b)
							Genotoxicity	Comet assay	Increase in tail length with 70 nm NPs	
SiNPs	HepG2	Human liver epithelial	7, 20, and 50 nm	Shanghai Cabot Chemical	20–640 µg/ml	24, 48 and 72 h	Cell viability	MTT assay	Dose and time-dependent reduction	Lu et al. (2011)
	LC-02	Human hepatocyte					Oxidative stress	DCFH-DA assay	Increase in HepG2	
							Oxidative stress	GSH depletion	Increase in HepG2	
							Apoptosis	Annexin V/PI staining	Dose-dependent increase in HepG2	
SiNPs;	Kupffer cells	Rat liver macrophage	15 nm	Sigma-Aldrich	50–800 µg/ml Supernatant was incubated with BRL cells	24 h	Cell viability	CCK-8 assay	Dose-dependent reduction in BRL	Chen et al. (2013)
	BRF	Rat liver cells					Cytotoxicity	LDH assay	Dose-dependent increase in BRL	
							Oxidative stress	DCFH-DA assay	Dose-dependent increase in KCs	
							Oxidative stress	GSH depletion	Dose-dependent increase in KCs	
							Pro-inflammatory response	ELISA	Dose-dependent increase of TNF-α in KCs	
							NO production	Griess reagent	Dose-dependent increase of NO in KCs	
							Cellular morphology	Phase contrast microscopy	Cell damage observed for BRL	
SiNPs	RAW 264.7	Mouse macrophage	12 nm	Sigma-Aldrich	200 and 400 µg/ml	24 h	Cell viability	WST-8 assay	Dose-dependent reduction	Hashimoto and Imazato (2015)
							Genotoxicity	Hoechst/PI staining	Deformation of nuclei at both concentrations	
							Genotoxicity	Comet assay	Dose-dependent increase	
							Genotoxicity	Micronuclei induction	Increase	
							Cellular uptake	SEM and TEM	NPs detected in vesicles and in nucleus	
SiNPs	Human peripheral lymphocytes	Human	10–20 nm	Sigma-Aldrich	50–100 µg/ml	24 h	Cell viability	MTT assay	Dose-dependent reduction	Rajiv et al. (2015)
							Cytotoxicity	LDH assay	Dose-dependent increase	
							Oxidative stress	DCFH-DA assay	Dose-dependent increase	
							Oxidative stress	LPO assay	Dose-dependent increase of MDA formation	
							Oxidative stress	GSH depletion	Dose-dependent increase	
							Oxidative stress	SOD assay	Dose-dependent decrease	
							Oxidative stress	Catalase assay	Dose-dependent increase	
							Genotoxicity	Comet assay	Increase	
							Chromosomal aberrations	Giemsa staining and microscopy	No effect	
SiNPs	Primary microglial cells	Rat brain macrophage like cells	20 nm	Sigma-Aldrich	250 and 500 mg/ml	24 h	Cell viability	MTT assay	No reduction	Xue et al. (2012)
	PC12	Rat neuron like cells					Pro-inflammatory response	ELISA	Mild increase of cytokines such as IL-6, TNF-α and IL-1β	
							NO production	Griess reagent assay	No effect	
							Nuclear binding activity	RT-PCR	No effect	
							Inflammatory factors	Western blot	No effect	
SiNPs	PC12	Rat neuron like cells	25± nm	Saint Louis	25–200 µg/ml	24 h	α-Synuclein levels	Western blot	Dose-dependent increase	Xie and Wu (2016)
							Cellular uptake	TEM	Increase	
							Autophagy	Western blot	Dose-dependent Increase of LC-II and Beclin 1	
							Pathway analysis	Western blot	Dose-dependent decrease of PI3K-Akt-mTOR signaling	
SiNPs	RAW 264.7	Mouse macrophage	10, 50, 300, and 1000 nm (with and without amine modification)	Micromod Partikeltechnologie	6.25–100 µg/m (with LPS and PGN)	24 h	Cell viability	WST-8	Dose and size-dependent reduction (only for bare SiNPs)	Uemura et al. (2016)
							Pro-inflammatory responses	ELISA	Dose-dependent increase of TNF-α and decrease of IL-6 (only for bare SiNPs). IL-6 decrease for micro particles	

*TEER* Transepithelial electrical resistance, *RT-PCR* Reverse transcription polymerase chain reaction, *ISDD* In vitro sedimentation, diffusion and dosimetry model, *FACS* Fluorescence-activated cell sorting, *LSCM* Laser scanning confocal microscopy, *AO/EB* Acridine orange/ethidium bromide, *FISH* Fluorescence in situ hybridization, *EGFR* Epidermal growth factor receptor, *KRAS* Ki-ras2 Kirsten rat sarcoma viral oncogene homolog, *HPRT* Hypoxanthine phosphoribosyl transferase, *HSCA* High content screening and analysis, *DAM-FM* 4-amino-5-methylamino-2′,7′-difluorescein, *PI* Propidium iodide, *Nrf 2* Nuclear factor erythroid 2-related factor 2

**Table 2:** In vivo studies on SiNPs toxicity

Type of SiNPs	Exposure route	Primary particle size	Source	Species and Strain	Exposure dose	Exposure duration	Organ(s)/sample(s)	Effect(s)/endpoint(s)	Assay (s)/method(s)	Results	References
C-SiNPs	Oral	20 and 100 nm	E&B Nanotech	Rat; SD (female and male)	500 and 1000 mg/kg bw	1 h–10 days	Animal	Clinical signs	Observation	No animal death	Lee et al. (2014)
							Lungs, liver, brain, kidneys, testis and spleen	Tissue distribution	Molybdenum blue method	NPs detected in lungs, liver, spleen and kidneys	
								NP Localization	TEM	NPs detected in the nuclei of hepatocytes	
							Urine and feces	Excretion kinetics	Molybdenum blue method	Most NPs excreted via feces	
C-SiNPs	Oral	20 and 100 nm (uncoated or l-arginine coated)	E&B Nanotech	Mice; C57BL/6	750 mg/kg bw	Daily for 14 days	Blood	WBCs count	Serum analyzer	Increase	Kim et al. (2014b)
								Pro-inflammatory response(s)	Multiplex analysis	Differential expression of cytokines	
							Spleen	Oxidative stress	DCFH-DA assay	Increase	
								Oxidative stress	SOD and GPx assay	No effect	
								Oxidative stress	Griess reagent/NO production	Increase	
C-SiNPs	Oral	12 nm	ABC Nanotech	Rat; SD	Acute-1959 and 2061; Sub-acute-489.8, 979.5, 1959; Sub-chronic: 244.9, 489.8 and 975.9 mg/kg bw	Acute—14 days Sub-acute—daily for 14 days Sub-chronic: daily for 13 w	Blood	Blood cell count	Hematoanalyzer	No effect	Yun et al. (2015)
								Organ damage bio-markers	Serum analyzer	No effect	
							Urine	Urinalysis	Urine analyzer	No effect	
							Eye	Ophthalmology	Morphological examination	No effect	
							Major organs	Tissue distribution	ICP-MS	NPs not detected	
							Urine and feces	Excretion kinetics	ICP-MS	Most NPs excreted via feces	
C-SiNPs	Oral gavage	20 and 80 nm, coated with l-arginine	E&B Nanotech	Rats; Crl:CD(SD)	500, 1000 and 2000 mg/kg bw	Daily for 90 days	Animal	Clinical signs	Observation	No animal death	Kim et al. (2014c)
							Blood	Blood cell count	Hematoanalyzer	No effect	
								Organ damage bio-markers	Serum analyzer	No effect	
							Eye	Ophthalmology	Observation of ocular fundus	No effect	
							All major organs	Necropsy	Weighing organs	No effect	
								Histopathology	H & E staining	No effect	
C-SiNPs	Intratracheal	9,15, 30 and 55 nm	AkzoNobel AB	Rat; Wistar (female)	360 µg in 500 ml of saline	3 days	Blood	Blood cell count	Hematoanalyzer	Increase of polymorphonulcear neutrophils and lymphocytes	Maser et al. (2015)
							Lungs	Histopathology	H & E staining	Mild increase of granulomatous inflammation	
								Genotoxicity	Comet assay	No effect	
							Bone marrow	Genotoxicity	Micronucleus test	No effect	
C-SiNPs	Dermal	20 nm, coated with l-arginine	E&B Nanotech	Rat; Sprague–Dawley (SD)	500 mg, 1000 mg, and 2000 mg/kg bw	Daily for 90 days	Animal	Clinical signs	Observation	No animal death	Ryu et al. (2014)
							Blood	Blood cell count	Hematoanalyzer	No effect	
								Organ damage bio-markers	Serum analyzer	No effect	
							All major organs	Necropsy	Organ weighing	No effect	
								Histopathology	H & E staining	No effect	
C-SiNPs	Dermal	20 and 100 nm (uncoated or l-arginine coated)	E&B Nanotech	Rat	2000 (coated) and 1000 mg/kg bw(uncoated)	Daily for 28 d	Brain	Blood–brain barrier damage	Evans blue staining	No effect	Shim et al. (2014)
								Tissue distribution	TEM-EDX	NPs not detected	
C-SiNPs	Intraperitoneal	50 nm	Polysciences	Mice; Male tuck ordinary	0.25 mg/kg bw	24 h	Blood	Blood cell count	Hematoanalyzer	Increase of leukocytes	Nemmar et al. (2016)
								Organ damage bio-markers	Serum analyzer	Increase of CK, ALT and AST	
							Lungs, heart, liver, kidneys, and brain	Oxidative stress	LPO, SOD and catalase assay	Increase	
								Pro-inflammatory response(s)	ELISA	Differential expression of IL-6, TNF-α and IL-1β	
								Genotoxicity	Comet assay	Increase	
C-SiNPs	Intravenous	64 nm	Laboratory synthesis	Mice; ICR (male and female)	0, 29.5, 103.5, and 177.5 mg/kg bw	14 days	Blood	Organ damage bio-markers	Serum analyzer	Increase of LDH, ALT and AST	Yu et al. (2013)
							Liver, spleen, kidneys, heart, lungs, and brain	Histopathology	H & E staining	Tissue damages observed	
								CD-68 positive cells	CD-68 staining	Increase in liver and spleen	
								NP localization	TEM	NPs detected in liver macrophages and in the endothelial cells of lungs and kidneys	
								Tissue distribution	ICP-OES	Si detected in liver and lungs	
S-SiNPs	Intratracheal	50 nm, with or without amine modification	Laboratory synthesis	Mice; C57BL/6 (male)	4 and 20 mg/kg bw	24 h	Lungs	Inflammation	BALF cell count and LDH assay	Dose-dependent increase of total cell number, macrophages, neutrophils and LDH release	Morris et al. (2016)
								Oxidative stress	ROS/RNS production	Dose-dependent increase	
S-SiNPs	Intratracheal	58 nm	Laboratory synthesis	Mice; C57 (male)	2 mg/kg bw	Once every 3 days for 45 days	Testis	Histopathology	H & E staining	Decrease in mature sperm and primary spermatocytes	Zhang et al. (2016)
								Meiotic regulating factors	Western blot	Cell cycle arrest observed	
								Oxidative stress	DCFH-DA assay	Increase	
							Sperm	Quality evaluation	Microscopy	No effect	
S-SiNPs	Intratracheal	43 nm	Laboratory synthesis	Mice; BALB/c(female)	0, 7, 21, and 35 mg/kg bw	Once every 3 days for 15 days	Lungs, liver and heart	Histopathology	H & E staining	Increase of Inflammation	Yang et al. (2016)
								Organ damage bio-markers	Serum analyzer	Increase of BUN, CREA, uric acid, and AST	
								NP localization	TEM	NPs detected in cytoplasm and lysosomes	
								Macrophage activation	Immunohistochemistry	Increase	
								Inflammatory response	Multiplex flow cytometry	Increase of IL-8, TNF-α and IL-6	Duan et al. (2014a, b)
S-SiNPs	Intravenous	62 nm	Laboratory synthesis	Mice; ICR	29.5, 103.5, and 177.5 mg/kg bw	14 days	Heart	Autophagy	LC3 and VEGFR2 positive staining	Increase of LC3	
									TEM	Increase of autophagic ultrastructures	
									Cell cytoskeleton staining	Weakening of F actin	
									MMP measurements	Dose-dependent decrease	
									LC3-II/LC3-I ratio	Increase	
M-SiNPs	Intragastrical	Spherical 83 nm, short rods (AR 1.75) and long rods (AR 5)	Laboratory synthesis	Mice; ICR (male)	40 mg/kg bw	14 days	Blood	Blood cell count	Hematoanalyzer	No effect	Li et al. (2015)
								Organ damage bio-markers	Serum analyzer	Increase of LDH release and CREA	
							Liver, kidneys, spleen, lungs and small intestine	Histopathology	H & E staining	Gross tissue damage in kidneys	
								Tissue distribution and excretion kinetics	ICP-OES and TEM	shape-dependent distribution and clearance in organs	
M-SiNPs	Intravenous	1.5 (short rods) and 5 (long rods) aspect ratio; standard or PEGylated	Laboratory synthesis	Mice	20 mg/kg bw	2 h, 24 h and 7 days	Blood	Blood cell count	Hematoanalyzer	No effect	Huang et al. (2011)
								Organ damage bio-markers	Serum analyzer	Increase of TBIL, CREA and BUN	
							Liver, spleen, lungs and kidneys	Histopathology	DAPI	Gross tissue damage in kidneys	
								Tissue distribution	ICP-OES	PEGylation reduced distribution in liver and spleen	
							Urine and feces	Excretion kinetics	TEM/EDX	Short rods cleared rapidly than long rods	
M-SiNPs	Intraperitoneal	~198 nm	Laboratory synthesis	Mice; BALB/C	150, 300, and 600 mg/kg bw	2 and 12 days	Blood	Organ damage bio-markers	Serum analyzer	Increase of AST, ALT BUN and CREA	Chen et al. (2015)
							Kidneys	Histopathology	H & E staining	Detection of renal interstitial fibrosis	
								Tissue damage	Masson’s trichrome staining	Dose and time-dependent increase in kidney injury	
Pr-SiNPs (NM 200 & 201), Py-SiNPs (NM 202 & 203)	Oral gavage	18–24 nm	JRC repository	Rat; SD (male)	5, 10, and 20 mg/kg bw	0, 24, and 45 h	Blood	Oxidative stress	LPO assay	No effect	Tarantini et al. (2015a, b)
							Liver, kidneys, spleen, intestine, blood and bone marrow	Histopathology	H & E staining	No effect	
								Genotoxicity	Alkaline, FpG modified comet assay and micronucleus test	No effect	
Pr-SiNPs (NM 200)	Oral gavage	10–15 nm	JRC repository	Rat; Wistar	100, 300, or 1000 mg/kg bw at a dose volume of 10 mL/kg bw	Daily for 14 days (from gestation period 6–19)	Animal	Clinical signs	Observation	No animal death	Hofmann et al. (2015)
								Body weight	Observation	No effect	
							Gravid uterus	Necropsy	Cesarean	No effect	
							Fetus	Fetus gross damage	Morphological examinations	No effect	
Py-SiNPs	Exposure via food	7 nm and NM 202 (10–25 nm)	JRC repository	Rat; SD	Sub-acute: 100, 1000,2500 mg/kg bw Sub-chronic: 2500 mg/kg bw	Sub-acute: daily for 28 days Sub-chronic: daily for 84 days	Blood	Organ damage bio-markers	Serum analyzer	No effect	Zande et al. (2014)
								Plasma IgG and IgM and cytokine analysis	ELISA	No effect	
							Liver	Transcriptomic analysis	mRNA quantification kit	No effect	
							Liver, jejunum, kidneys and spleen	Histopathology	H & E staining	Presence of fibrosis in the liver of NM 202 treated rats	
							Liver, spleen, lungs, brain and testis	Tissue distribution	ICP-MS	NM 202 detected in lungs, kidneys and spleen; SAS only in the spleen	
Py-SiNPs	Intravenous	13 ± 5 nm	Vekton Ltd	Rat; Wistar	7 mg/kg bw	7, 30 and 60 days	Blood	Hemodynamics	Blood pressure measurement and heart rate	No effect	Zhuravskii et al. (2016)
								Blood cell count	Hematoanalyzer	No change	
								Organ damage bio-markers	Serum analyzer	Increase of ALP at 7 d	
							Liver, heart, spleen, lungs and kidneys	Tissue distribution	Atomic absorption spectrometry	Si detected in liver, lungs and spleen	
								NP localization	SEM	NPs detected in hepatocytes	
								Histopathology	H & E staining	Induction of fibrosis	
SiNPs	Oral gavage	70 nm, 300 and 1000 nm with or without carboxyl or amine groups	Micromod Partikeltechnologie	Mice; BALB/c	2.5 mg/mouse	Daily for 28 days	Blood	Blood cell count	Hematoanalyzer	No effect	Yoshida et al. (2014)
								Organ damage bio-markers	Serum analyzer	No effect	
							All major organs	Histopathology	H & E staining	No effect	
							Intestine	Intestinal absorption	Evented gut sac analysis	Absorption of coated 70 nm SiNPs	
SiNPs	Oral gavage	10–15 nm	TECNAN	Rat; Wistar (male)	333.3 mg/kg bw	Daily for 5 days	Animal	Clinical signs	Observation	Symptoms of vomiting and severe lethargy	Hassankhani et al. (2014)
							Blood	Organ damage bio-markers	Serum analyzer	Increase	
							Kidneys, lungs and testis	Histopathology	H & E staining	Tissue damage observed	
SiNPs	Intratracheal	Three SiNPs (30, 60, and 90 nm) and one fine-sized silica (600 nm)	Laboratory synthesis	Rat; Wistar (male)	2,5 and 10 mg/kg bw	Daily for 16 days	Blood and Heart	Blood cell count	Hematoanalyzer	Increase of WBCs & platelets and decrease of hemoglobin &RBCs	Du et al. (2013)
								Oxidative stress	LPO, GSH, SOD and GSH-Px assay	Increase of MDA formation	
								Oxidative stress	NO/NOS	Increase of NO and decrease of NOS	
								Pro-Inflammatory response(s)	ELISA	Increase of TNF-a, IL-1b and IL-6	
								Tissue distribution	ICP-OES	NPs detected in heart	
SiNPs in paints	Oropharyngeal	19 nm	SiNPs	Mice; BALB/c mice	20 µg/aspiration	Once a week for 5 w	Lungs	Inflammation	BALF Cell count	Increase of macrophages and neutrophils	Smulders et al. (2014)
								Pro-inflammatory response(s)	ELISA	No effect	
								Tissue distribution	ICP-MS	NPs detected in lungs	
SiNPs	Intranasal	10 and 80 nm SiNPs	Nanoamor	Rat; Wistar (male)	150 µg/50 µl PBS/rat	Daily for 30 days	Brain	Oxidative stress	LPO assay	Increase of MDA formation	Parveen et al. (2015)
									Xylenol orange assay	Increase of H_2_O_2_ levels	
									GSH depletion	Increase	
									SOD, CAT and GPx levels	Increase	
								Pro-Inflammatory response(s)	RT-PCR and ELISA	Increase TNF-α, IL-1β and MCP-1	
								Nuclear binding activity	Immuno blot analysis	Increase	
								Tissue distribution	ICP-OES	Si detected in frontal cortex, corpus striatum and hippocampus	
SiNPs	Topical	70 nm, 300 and 1,000 nm	Micromod Partikeltechnologie	Mice; BALB/c	250 mg/ear	Daily for 28 days	Skin	Apoptosis	TUNEL staining	Increase	Nabeshi et al. (2011b)
							Animal	Tissue distribution	In vivo Imaging	70 nm SiNPs detected around the liver	
	Intravenous				30 mg/kg bw	24 h					
								NP internalization	TEM	70 nm SiNPs detected in cytoplasm and nucleus of the parenchymal hepatocytes (liver)	
SiNPs	Intravenous	15 nm	Sigma-Aldrich	Rat; SD (male)	50 mg/kg bw	48 h	Blood	Blood cell count	Hematoanalyzer	Increase of WBC, lymphocytes, monocytes and neutrophils	Chen et al. (2013)
							Liver	CD-68 positive cells	CD-68 staining	Increase	
								Oxidative stress	GSH and SOD assay	Increase	
								Injury bio-markers	Proton-NMR spectroscopic analysis	Increase	

*JRC* Joint research commission, *TEM* Transmission electron microscopy, *WBCs* White blood cells, *ICP-MS* Inductively coupled plasma mass spectrometry, *H & E* Hematoxylin and eosin, *EDX* Energy dispersive X-ray spectroscopy, *ELISA* Enzyme linked immuno sorbent Assay, *CD* Cluster of differentiation, *ICP-OES* Inductively coupled plasma optical emission spectroscopy, *BALF* Bronchoalveolar lavage fluid, *VEGFR* Vascular endothelial growth factor receptor, *MMP* Mitochondrial membrane potential, *DAPI*-4′,6-diamidino-2-phenylindole, dilactate, *SEM* Scanning electron microscopy, *TUNEL* Terminal deoxynucleotidyl transferase dUTP nick end labeling, *NMR* Nuclear magnetic resonance

## In vitro studies

### Cytotoxicity

Oxidative stress (over production of reactive oxygen species, i.e., ROS) induced by NPs could damage the cellular components and lead to cell death via apoptosis (Fu et al. 2013). Therefore, studies reporting on cytotoxicity and oxidative stress were summarized in this section.

#### Cytotoxicity associated with oxidative stress

Duan et al. (2013a) showed that S-SiNPs (62 nm) induced time- (6, 12, and 24 h) and dose-dependent (25–100 µg/ml) reduction in cell viability (assessed by 3-(4,5-dimethylthiazol-2-Yl)-2,5-diphenyltetrazolium bromide, i.e., MTT), loss of membrane integrity (lactate dehydrogenase (LDH) release) and apoptosis (Annexin V/PI staining) in human umbilical vein endothelial cells (HUVECs). Apoptosis was also induced in lung (A549) and skin epithelial cells (A431) treated with Pr-SiNPs (15 nm). A dose-dependent increase (25–200 µg/ml for 72 h) in cytotoxicity (MTT and LDH), ROS production (assessed by dichlorodihydrofluorescein assay, i.e., DCFH-DA), lipid peroxidation (measurement of malondialdehyde, i.e., MDA), and apoptosis (caspase 3 and 9 activity) was observed in both cell lines. The lung cells showed, in general, a slightly higher toxic response compared to skin cells (Ahamed 2013).

SiNPs (20 and 80 nm) induced P53-mediated apoptosis in human fetal lung fibroblasts (HFL-1). At the dose of 500 µg/ml, 20 nm SiNPs induced a threefold increase in DCF fluorescence compared to 80 nm. In addition, increased expression of P53, upregulation of cytochrome C (CytC) and caspase 9, and downregulation of anti-apoptotic protein B-cell lymphoma 2 (bcl2) was observed in cells treated with 1000 µg/ml for 48 h (Xu et al. 2012). Another study with lung fibroblasts also showed that SiNPs (20 nm) could reduce cell viability (MTT) by inducing apoptotic cell death (fluorescence microscopy) in a dose-dependent manner (250–1000 µg/ml for 48 h) (Zhang et al. 2011). Athinarayanan et al. (2014) isolated SiNPs (10–50 nm) from commercial food products processed with food additive silica (E551) and exposed human lung fibroblasts (WI-38 cell line) with increasing doses (25–400 µg/ml). After 24 h, they observed cytotoxicity (MTT) in a dose-dependent manner and ROS production (DCFH-DA) at 50 µg/ml.

#### Cytotoxicity not associated with oxidative stress

Py-SiNPs (12 and 40 nm) induced a significant size and dose- (31.3, 93.8, and 156.3 µg/cm^2^ culture well) dependent cytotoxicity (LDH, Sulphorodhamine B assay (SRB) and water-soluble tetrazolium-1(WST-1)) in human colon carcinoma cell line (HT29), while no induction of ROS (DCFH-DA) was observed (Gehrke et al. 2013). In the study by Napierska et al. (2012a), 50 μg/ml (24 h) of 16 nm iron-doped S-SiNPs and pure S-SiNPs induced strong cytotoxicity (MTT and LDH) in a human endothelial cell line (EA.hy926), but a significant increase in oxidative stress markers [GSH depletion, malondialdehyde (MDA formation), induction of heme oxygenase-1, glutathione reductase, and NADPH oxidase-1] was observed only for iron-doped SiNPs.

#### Conclusion: cytotoxicity

Cytotoxicity of SiNPs was investigated using different cell lines and incubation times, making the comparison between studies difficult. However, from Table 3, it is clear that all types of SiNPs induced cytotoxicity. Significant (compared to untreated cells) cytotoxic effects were observed only at or above the concentration of 25 µg/ml. Furthermore, it can be clearly seen that SiNPs induced oxidative stress and mediated apoptosis mainly via the *intrinsic or mitochondrial pathway* (caspase-dependent pathway) in a size- and dose-dependent manner. ROS-mediated toxicity is believed to be an important mechanism of NP toxicity including SiNPs (Manke et al. 2013). Nevertheless, Py- and S-SiNPs caused cytotoxicity without measurable levels of ROS production. It was demonstrated that the *disturbance of membrane integrity* due to direct cell-membrane interaction might be another possible mechanism of NP cytotoxicity (Fröhlich et al. 2009; Thomassen et al. 2011). However, neither of these studies did substantiate these observations and, therefore, SiNPs cytotoxic effects in the absence of oxidative stress remain poorly understood.

**Table 3:** Comparison of toxic effects induced by different types of SiNPs (in vitro)

SiNP type	Cell type	Cytotoxicity	Apoptosis	Genotoxicity	Oxidative stress	Pro-inflammation	References
Colloidal	Caco2	✓	✓	✓	✓	✓	Tarantini et al. (2015a, b)
Colloidal	HepG2	✓	✓	✓	✓	n/a	Li et al. (2011)
Colloidal	V79 and A549	✓	n/a	✓	n/a	n/a	Maser et al. (2015)
Colloidal	J744.1	✓	✓	n/a	✓	✓	Lee et al. (2011)
Colloidal	PBMC	✓	✓	n/a	✓	✓	Mendoza et al. (2014)
Stöber	Huvecs	✓	✓	✓	✓	✓	Duan et al. (2013a)
Stöber	HepG2	✓	✓	n/a	✓	n/a	Sun et al. (2011)
Stöber	HepG2	✓	✓	n/a	✓	n/a	Wang et al. (2013)
Stöber	HaCaT	✓	✓	n/a	✓	n/a	Liang et al. (2014)
Stöber	EA.hy926	✓	n/a	n/a	✓	n/a	Napierska et al. (2012a, b)
Precipitated	V79	✓	✓	✘	✘	n/a	Guichard et al. (2016)
Precipitated	Mouse fibroblast	✓	n/a	✘	n/a	n/a	Uboldi et al. (2012)
Precipitated	GES-1 and caco2	✓	✓	n/a	✓	n/a	Yang et al. (2014a, b)
Precipitated	HepG2	✓	✓	n/a	✓	n/a	Ahmad et al. (2012)
Precipitated	A549 and A431	✓	✓	n/a	✓	n/a	Ahamed (2013)
Precipitated	M-HS	✓	n/a	n/a	✘	✓	Di Cristo et al. (2016)
Precipitated	RAW.264.7	✓	n/a	n/a	✘	✓	Di Cristo et al. (2016)
Pyrogenic	V79	✓	✓	✓	✘	n/a	Guichard et al. (2016)
Pyrogenic	GES-1 and caco2	✓	✓	n/a	✘	n/a	Yang et al. (2014a, b)
Pyrogenic	HT-9	✓	n/a	n/a	✘	n/a	Gehrke et al. (2013)
Pyrogenic	RAW.264.7	✓	n/a	n/a	✘	✓	Di Cristo et al. (2016)
Pyrogenic	M-HS	✓	n/a	n/a	✓	✓	Di Cristo et al. (2016)

n/a, not investigated; ✓, positive; ✘, negative

**Fig. 2:**
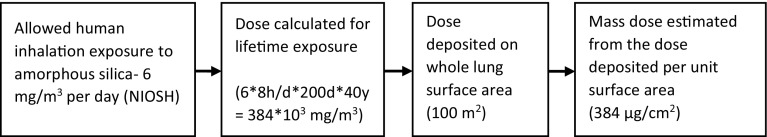
In vitro dose estimation from human tolerable levels (OELs)

Furthermore, some authors used very high concentrations that may cause “overloading” of cells and modify the nature of NP–cell interactions (Wittmaack 2011). In these cases, it is difficult to evaluate whether the observed effects are physiologically relevant. Although it is challenging, we consider a dose of 384 µg/cm^2^ or higher as irrelevant to human inhalation exposure for amorphous silica, based on the estimation that can be derived from the occupational exposure levels (OELs) (Fig. 2).

### Genotoxicity

In this section, we presented studies on genotoxic effects of SiNPs as it is used as another major endpoint to characterize hazard of NMs. Direct interaction with DNA, oxidative DNA damage, depletion of anti-oxidants, cell cycle arrest, and abnormal expression of genes have been identified as potential mechanisms of NP mediated (geno)toxicity (Donaldson et al. 2010).

#### DNA damage associated with oxidative stress

Exposure to SiNPs (15, 30, and 100 nm) resulted in a size- and dose- (2.5–10 µg/ml for 24 h) dependent increase in 8-hydroxy-2′-deoxyguanosine levels (8-OH-dG), phosphorylation of histone on serine-139 (ɣH2AX), and DNA strand breaks (comet) in human keratinocytes (HaCaT) (Gong et al. 2012). Nabeshi et al. (2011a) also demostrated that exposure to SiNPs (70 nm; 10–90 µg/ml for 24 h) resulted in the increase of oxidative DNA damage (8-OH-dG levels) in HaCaT cells. SiNPs were taken up via actin-mediated endocytosis. Micron-sized particles used in these studies showed no or little effects.

The viability of human Caucasian colon adenocarcinoma (Caco-2) cells dropped to 40% when exposed to 15 nm C-SiNPs (64 µg/ml for 24 h), and, at this same concentration, nearly a threefold increase in micronuclei formation, fivefold increase in histone phosphorylation (ɣH2AX), and a significant increase in DCF fluorescence were observed. The particles were localized within lysosomes and endocytic compartments, but not in the nucleus. 55 nm C-SiNPs did not induce any of these effects at the same concentration (Tarantini et al. 2015b).

A non-significant increase in % tail DNA (comet assay) and no chromosomal aberrations were induced by 17 nm SiNPs in human peripheral lymphocytes treated with 100 µg/ml, while a dose-dependent (50–100 µg/ml for 24 h) ROS production (DCFH-DA) and GSH depletion were observed (Rajiv et al. 2015).

#### Cell cycle arrest associated with oxidative stress

S-SiNPs (62 nm) induced increase in DCF fluorescence and decrease in superoxide dismutase (SOD) and glutathione peroxidase (GSH-Px) in HUVECs in a dose-dependent manner (25–100 µg/ml for 24 h). Oxidative stress was linked to cell cycle arrest at G2/M checkpoint (upregulation of chk 1 and downregulation of Cdc25c, Cyclin B1, and Cdc2) and increase in apoptosis (Duan et al. 2013a). In the study by Li et al. (2011), a size-dependent (19, 43, and 68 nm) increase in oxidative stress (DCF fluorescence) and cell cycle arrest in S and G2/M was observed in HepG2 cells exposed to 100 µg/ml of C-SiNPs. Cell cycle arrest in G2/M phase along with the increase in ROS was also noticed in human hepatic cell line (LC-02) treated with S-SiNPs (50 nm) in a dose-dependent manner (50–200 µg/ml for 24 h) (Wang et al. 2013).

#### DNA damage not associated with oxidative stress

Genotoxicity of Py-SNP (20 and 25–70 nm), Pr-SNP (20 nm), and C-SNP (15 and 40–80 nm) SiNPs were studied in Chinese hamster lung fibroblasts. Py-SiNPs (20 nm) induced a significant increase in DNA strand breaks at 66 µg/ml (24 h), while C-SiNPs (15 nm) showed a similar effect only at 252 µg/ml. Neither of these SiNPs did induce ROS. SiNPs in the size range 25–80 nm exerted no or little genotoxicity (Guichard et al. 2016).

#### Genotoxicity reports without the assessment of oxidative stress

M-SiNPs (100 nm) induced a significant increase in phosphorylated-ɣH2AX-foci in HT-29 cells treated with a dose of 10 µg/ml for 24 h (Sergent et al. 2012). In the human embryonic kidney cell line (HEK293), 579 genes were upregulated and 1263 genes were downregulated after 24 h of exposure (100 µg/ml) to 100 nm M-SiNPs (Zhang et al. 2015). In another study, 15-nm C-SiNPs induced a significant increase in DNA strand breaks (comet assay) in chinese hamster cells (V79) and A549 cells at 100 µg/ml (24 h), but, for 55 nm, this effect was observed only in A549 cells (Maser et al. 2015). A significant increase in DNA tail length (comet assay) was observed in HaCaT cells treated with 30 µg/ml (24 h) of 70 nm SiNPs (Nabeshi et al. 2011b).

Exposure to C-SiNPs (~7 nm) resulted in positive genotoxic effects (Lymphoma assay) in mouse lymphoma cells treated with 100 and 150 µg/ml for 4 h (Demir and Castranova 2016). SiNPs (12 nm) induced DNA strand breaks in RAW 264.7 at 200 and 400 µg/ml, but the induction of micronuclei was noticed only at 400 µg/ml. The particles were internalized in vesicles and in the nucleus (Hashimoto and Imazato 2015).

At any tested concentrations (1–100 µg/ml for 72 h), SiNPs (10–25, 5–30, 35, 15, 80, and 90 nm) neither induced cytotoxicity nor micronuclei in immortalized Balb/3T3 fibroblasts (Uboldi et al. 2012). In another study, Pr-SiNPs (NM-200 and NM-201) and Py-SiNPs (NM-202 and NM-203) with primary size between 14.5 and 16 nm did not induce any micronuclei (cytokinesis block micronucleus assay) in human peripheral lymphocytes exposed to different concentrations (200–1250 µg/ml) over 24 h (Tavares et al. 2014). It is also worthy to note that, in the latter study, the positive control used did not differ from control conditions.

#### Conclusion: genotoxicity

C-SiNPs and S-SiNPs induced genotoxicity in human tumor cell lines (lung, kidney, skin, and gastro-intestinal systems) and the amplitude of the effect negatively correlated with the size of the NPs. DNA strand breaks were observed at low concentrations (2.5–10 µg/ml), particularly in skin-derived cell lines. The genotoxic effects of C- and S-SiNPs were mainly associated with the induction of oxidative stress, while such information is very limited for other types (Py- and Pr-SiNPs). One study indicated that Py-SiNPs induce DNA damage without the generation of ROS, suggesting that other mechanisms such as direct DNA damage might be involved (Magdolenova et al. 2014). However, it is very difficult to judge whether such genotoxic effect is direct or indirect, since the cellular uptake and subcellular localization of SiNPs are not often reported. Furthermore, several factors such as SiNP properties, cell type, and exposure scenarios (such as concentrations, assays, and endpoints) may influence the outcomes (Magdolenova et al. 2014), making the comparison difficult between studies and indicating an urgent need for the standardization of genotoxicity studies.

### Immunotoxicity

NPs entering the body will most probably interact with immune cells, as they are the first line of defence in human body. In this section, we presented the immune responses induced by SiNPs in different cell lines.

#### Immunotoxicity associated with oxidative stress

Hara et al. (2014) exposed THP-1-derived macrophages to 100 µg/ml of SiNPs (30 nm) for 6 h and found a significant increase in interleukin-1-beta (IL-1β), ROS production, and SiNP uptake via phagocytosis. In the study of Choi et al. (2010), larger sized SiNPs (150–200 nm) were effectively phagocytosed by primary rat microglial cells after 24 h of exposure to different concentrations (0.0728–7.28 µg/ml). A significant increase in ROS, reactive nitrogen species (RNS) and IL-1β was detected at all concentrations.

#### Immunotoxicity not associated with oxidative stress

At 10 and 20 µg/ml, Di Cristo et al. (2016) found that Py-SiNPs (~14 nm) induced a stronger increase of tumor necrosis factor-alpha (TNF-α), interleukin(IL)-6, and IL-1β in RAW.264.7 macrophages compared to similar sized Pr-SiNPs; Notably, no SiNPs induced ROS in RAW.264.7 macrophages.

#### Immunotoxicity reports without the assessment of oxidative stress

A significant increase in TNF-α, IL-6, and IL-1α, mitogen activated protein kinases (MAPKs), and nuclear factor (NF)-κB were observed only for C-SiNPs (100 nm) in J774A.1 macrophages exposed to 100 µg/ml of same sized (100 nm) C-SiNPs or M-SiNPs (Lee et al. 2011).

Uemura et al. (2016) showed that SiNPs (10 and 50 nm) caused dose-dependent (6.25–100 µg/ml) increase in the production of TNF-α and decrease of IL-6 in RAW.264.7 macrophages, while their amine surface-modified counterparts did not. Furthermore, 300 and 1000 nm micron-sized particles (both bare and amine modified) also showed a dose-dependent decrease of IL-6. Notably, the effects were stronger for 50 nm compared to other particles. The same cell line was utilized by Yu et al. (2011) to investigate phagocytosis using inductively coupled plasma mass spectroscopy (ICP-MS), and they found that S-SiNPs (25 nm) were phagocytosed at least ten times more than M-SiNPs of same size and high aspect ratio SiNPs (AR 2, 4, and 8). In the study by Napierska et al. (2012b), THP-1 cells dosed with 5 µg/cm^2^ S-SiNPs (2 nm) showed a significant increase of IL-8, TNF-α, and macrophage inflammatory protein (MIP)-1α, while only a non-significant increase in MIP-1α expression was observed for 16 and 104 nm S-SiNPs.

#### Conclusion: immunotoxicity

The main cells used to study immune responses to SiNPs were ‘innate’ cells such as monocytes and macrophages. Therefore, the identified in vitro studies only address a very limited part of the immune system, essentially pro-inflammatory responses and potential phagocytosis. Furthermore, the data on immune responses and oxidative stress are very limited and, therefore, no firm conclusions can be made. SiNPs, not only induced stronger pro-inflammatory responses compared to sub-micron and micron sized particles but also size-specific effects within the nano-range in immune cells are observed. Besides size, shape and porosity seem to influence the phagocytosis of SiNPs.

### Autophagy

Recently, a growing body of evidence identified autophagy as a cellular defence mechanism against NP toxicity, since it plays a key role in removing misfolded proteins and clearing damaged organelles (Glick et al. 2010). Hence, we present here studies that show induction of autophagy upon exposure to SiNPs.

#### Autophagy associated with oxidative stress

The same S-SiNPs (62 nm) were used in three studies to investigate the induction of autophagy. Along with the dose-dependent (25–100 µg/ml) increase in ROS production, increase in autophagy bio-marker-microtubule-associated protein 1A/1B-light chain 3 (LC3) and monodansylcadaverine (MDC) labelled autophagic vacuoles were detected in HepG2 cells treated with 62 nm S-SiNPs. In addition, transmission electron microscopy (TEM) images revealed that autophagosomes and autolysosomes induced in the presence of SiNPs (Yu et al. 2014). The same S-SiNPs (62 nm) induced increase of LC3-II/LC3-I ratio and decrease of p-mTOR/mTOR, p-P13 K/P13 K and p-Akt/Akt in HUVEC cells in a dose-dependent (25–100 µg/ml) manner (Duan et al. 2013b, 2014a, b). The results of Guo et al. (2016) also suggest that 50 µg/ml of S-SiNPs (58 nm) could induce autophagy via MAPK/Bcl-2 and PI3K/Akt/mTOR signaling in HUVECs.

After 4 h of exposure to 200 µg/ml of S-SiNPs (50 nm), autophagosomes and ROS production was observed in HaCaT cells. The TEM images revealed that SiNPs were in the cytoplasm and lysosomes, but not in the nucleus. (Liang et al. 2014).

SiNPs (4–13 nm, 62.5 µg/ml) induced a time-dependent (24, 48, and 72 h) reduction in cell viability and increase in oxidative stress (DCF fluorescence and GSH depletion) in the lung fibroblast cell line MRC-5. Compared to control, a significant increase of autophagic vacuoles and LC-3 II/LC3-I ratio was also observed in a time-dependent manner (Voicu et al. 2015). A549 cells, when exposed to 100 and 1000 µg/ml of 20-nm SiNPs, showed threefold and fivefold increase in MDC fluorescence, respectively. In addition, autophagy genes such as ATG-12 and BECN were significantly upregulated (30- and 50-fold, respectively) along with increased production of ROS in cells dosed with 1000 µg/ml (Nowak et al. 2014).

#### Conclusion: autophagy

SiNPs, particularly S-SiNPs induced autophagy mainly via oxidative stress-mediated upregulation of autophagy-related genes and differential regulation of Akt/mTOR signaling. Similar to cytotoxicity, 25 µg/ml appeared to be the lowest exposure concentration at which SiNPs exhibited significant effects. Furthermore, induced autophagy is correlated to cytotoxicity, suggesting that exposure to SiNPs caused irreversible (serious) cellular damage and resulted in autophagic cell death. Besides autophagy induction, lysosomal and autophagy dysfunction could be a potential mechanism of NPs toxicity (Stern et al. 2012), which has, however, not been investigated for SiNPs.

### Toxic effects on blood cells and endothelial dysfunction

Several studies suggest that NPs, when inhaled or ingested, can translocate across barriers (such as air–blood) of the body, enter the circulation, and interact with the cardiovascular system. In this section, we summarized the studies that report the effects of SiNPs on blood and endothelial cells.

#### Toxic effects on blood cells

Nemmar et al. (2015) showed that mouse blood platelets could aggregate after 3 min of incubation with 5 or 25 µg/ml of 50-nm C-SiNPs, while in the study of Jose Corbalan et al. (2012), such aggregation was observed in 15 min (10 µg/ml of 10 nm C-SiNPs). The latter study also showed fourfold reduction of the nitric oxide (NO)/peroxynitrite (ONOO^−^) ratio compared to non-treated platelets.

Maurer-jones et al. (2010) investigated the role of porosity of SiNPs on blood cell toxicity. M-SiNPs (25 nm) reduced the cell viability of red blood cells (RBCs) to 50% at the concentration of 270 µg/ml, while non-porous S-SiNPs of similar size required only 20 µg/ml to reach this level of cytotoxicity. In another study, 10% hemolysis (LC_10_) of RBCs was observed at 36 µg/ml for S-SiNPs (115 nm), while M-SiNPs required 154 µg/ml to induce the same effects. For amine-coated counterparts, LC_10_ were 97 and 30 µg/ml for S- and M-SiNPs, respectively (Yu et al. 2011).

#### Endothelium dysfunction

Exposure to different concentrations (12.5–100 µg/ml) of S-SiNPs (58 nm) resulted in a dose-dependent increase in inflammatory mediators such as IL-1β, IL-8, and TNFα, intercellular adhesion molecule-1 (ICAM-1), vascular cell adhesion molecule (VCAM-1), and monocyte chemoattractant protein-1 (MCP-1) in HUVEC cells (Guo et al. 2015). In the study of Corbalan et al. (2011), 10 µg/ml of 10-nm C-SiNPs induced extremely low NO/NOO^−^ ratio (~0.1) in HUVEC cells. Furthermore, free radical production, pro-inflammatory cytokines (IL-6 and IL-8), and NF-κB-binding activity were significantly increased in treated cells. In these two studies, increase in ROS was observed at all tested concentrations.

S-SiNPs (62 nm) induced an imbalance in the ratio NO/nitric oxide synthase (NOS) enzyme in HUVEC cells and such imbalance resulted in a significant increase of pro-inflammatory response (c-reactive protein CRP, IL-1β, IL-6, and TNFα) in a dose- (50–100 µg/ml) dependent manner (Duan et al. 2014b).

#### Conclusion: Toxic effects on blood cells and endothelial dysfunction

Endothelial cells and platelets together play a key role in maintaining the vascular homeostasis (Rajendran et al. 2013). C-SiNPs induced oxidative stress and disturbed NO/NOO^−^ ratio, which resulted in the aggregation of platelets and endothelial dysfunction. This information is not available for other types of SiNPs. Furthermore, C-SiNPs mediated endothelial dysfunction resulted in pro-inflammatory signals via the secretion of cytokines and adhesion molecules. Together, these results suggest the potential of SiNPs to cause vascular thrombosis and atherosclerosis (Radomski et al. 2005). Furthermore, SiNPs caused hemolysis of RBCs in a size-, charge-, and porosity-dependent manner.

### Neurotoxicity

NPs of very small size are capable of translocating across the blood–brain barrier (Hu and Gao 2010). Therefore, studies investigating effects on cells relevant for neurotoxicity are presented here.

Rat medulla tumor cells (PC12 cell line) incubated with the supernatant of 20 nm SiNPs-treated-microglial cells (250 µg/ml and 500 µg/ml for 24 h) did not show any effects compared to the control. Earlier in this study, no secretion of bio-mediators was observed in SiNPs-treated-microglial cells (Xue et al. 2012). In contrast to this study, PC12 cells exposed directly to SiNPs (25 nm; 25–200 µg/ml for 24 h) showed increased uptake and a dose-dependent increase in the induction of autophagy (increase in LC-II and Beclin 1) and inhibition of PI3 K-Akt-mTOR signaling (Xie and Wu 2016). Yang et al. (2014a) showed that exposure to SiNPs (15 nm; 10 µg/ml for 24 h) induced pathological signs of Alzheimer’s disease such as altered expression of amyloid precursor protein (APP) and neprilysin, enhanced phosphorylation of tau at Ser262 and Ser396, and activation of glycogen syntheses kinase (GSK)-3β in human SK-N-SH and mouse neuro2a neuroblastoma cells.

#### Conclusion: neurotoxicity

In vitro studies used cell lines of CNS-based cells, mainly neuron like cells. Although the data on the neurotoxic effects of SiNPs are very limited, studies above suggest that SiNPs can induce adverse effects including the markers of Alzheimer’s disease, when in direct contact with neuroblastoma cells.

### Miscellaneous issues

#### Influence of cell lines on SiNPs cytotoxicity

A dose- (80–640 µg/ml) dependent decrease in the viability and increase of apoptosis were observed in HepG2 cells in presence of SiNPs (7 and 20 nm), but a significant reduction was observed in normal human liver cells (LC-02) only at the unrealistic dose level of 640 µg/ml (Lu et al. 2011). In another study, SiNPs (10–50 nm) induced a dose-dependent (100–600 µg/ml) increase in LDH release in Caco-2 cells, but a significant release of LDH was observed only at the unrealistic dose of 600 µg/ml in human gastric epithelial cells (GES-1). Furthermore, exposure to these SiNPs (200 µg/ml for 48 h) also induced cell cycle arrest in S phase for GES cells and G2/M in Caco-2 cells (Yang et al. 2014b). In a porcine kidney cell line (LLC PK1) exposed to 20-nm SiNPs, a dose- (5–50 µg/ml) dependent increase in DCF fluorescence and MDA formation was observed, whereas human kidney cells (HK-2) showed little effects at 50 µg/ml (Passagne et al. 2012).

#### Conclusion: miscellaneous issues

No firm conclusions can be drawn from these cases; however, the cytotoxicity of SiNPs appears to vary with species and cell line.

#### Physiologically relevant cultures

##### Lung co-culture models

Co-cultures of lung cells are usually made with epithelial cells on the apical and endothelial cells on the basal compartment of a transwell membrane, with or without monocytes on top of the epithelial cells. In a co-culture (A 549 at the apical and ISO-HAS-1 at the basolateral compartment) exposed to 100-µg/ml 30-nm C-SiNPs (coated with or without surfactant), nearly a fivefold increase of IL-8 release for both forms of SiNPs was observed in both compartments (Kasper et al. 2015). When other epithelial cells were used—H441 cells—at the apical together with ISO-HAS-1 cells at the basolateral compartment, these C-SiNPs induced IL-8 were expressed in both compartments, while SiCAM-1 (6–600 µg/ml) and IL-6 (at 6 and 60 µg/ml) were observed only in the apical part (Kasper et al. 2011). The same co-culture model was exposed to 100 µg/ml of S-SiNPs (15, 35, and 80 nm) and the authors noticed an increase of IL-8, TNF-α, and surfactant protein (SP-A1 and SP-A2) expression compared to the control. In addition, less IL-8 and surfactant protein expression, and more TNF-α were observed in the co-culture added with THP-1, notably the effect was the highest for 35 nm (Farcal et al. 2012).

Napierska et al. (2012b) tested SiNPs with primary size 2, 16, 60, and 104 nm (dosed at 10 µg/cm^2^) in a co-culture (A549 at the apical and EA. hy926 at the basolateral compartment) and observed increase in cytokines such as IL-6, IL-8, TNF-α, and MIP-1α only for 60 and 2 nm (except IL-8). When THP-1 were added to the co-culture, a significant increase in IL-8 and decrease in TNF-α were observed only for 2 nm. The expression of cytokines was also differentially regulated for 16 and 104 nm before and after THP-1 added, but the effects were stronger for 60 and 2 nm, particularly 60-nm NPs.

##### Air–liquid interface

At the air–liquid interface (ALI), aerosolized and deposited 12-nm Py-SiNPs (52 µg/cm^2)^ and 50-nm S-SiNPs (117 µg/cm^2^) induced significantly less biological effects (LDH leakage, IL-8 release, COX-2 expression, and p38 phosphorylation) in A549 cells compared to A549 exposed to 15.6 µg/cm^2^ under sub-merged conditions (Panas et al. 2014).

#### Conclusion: physiologically relevant cultures

Sub-merged (co) cultures and ALI systems (Lenz et al. 2013; Panas et al. 2014) have been claimed to more closely mimicking the in vivo exposure scenarios compared to monocultures. In these systems, the toxicity and pro-inflammatory responses are significantly modulated by SiNPs, which represent the complexity of in vivo systems and need for the establishment of physiologically relevant in vitro cultures. However, at this moment, it is difficult to know whether these biological responses were influenced by SiNPs physico-chemical properties.

#### Chronic in vitro studies

In vitro chronic Py-SiNPs (12 nm) exposure of intestinal epithelial cell line (C2BBe1) was examined in a recent study. The cells were exposed to 10-µg/cm^2^ SiNPs for 24 h. After 24 h, the medium was replaced (without NPs) and cells were allowed to grow for 4–6 days. At the end of incubation, cells were passaged and again exposed for 24 h and grown for 4–6 days; this cycle was repeated for 29 passages (total life span). The cells and supernatants were collected at the end of each passage for analysis. Though the particles were internalized (only in a fraction of cells), no significant induction of necrosis, apoptosis, and LDH release and decrease in cell viability was observed for any of these conditions (McCracken et al. 2013).

## In vivo studies

### Ingestion exposure

#### Single exposure

Lee et al. (2014) investigated the tissue distribution and excretion profiles in Sprague–Dawley (SD) rats orally administered with a very high dose of 500 or 1000-mg/kg bw of C-SiNPs (20 and 100 nm). Silicon (Si) levels were significantly elevated in liver, kidney, lung, and spleen at 6-h post-administration, while no such increase was noticed in brain, ovaries or testes, esophagus, stomach, and intestine even after 7 days of post-administration. Nearly 75–80% of administered SiNPs were excreted via urine and 7–8% via feces. The author also noticed that 20 nm SiNPs excreted faster than the 100 nm SiNPs. In another study, C-SiNPs (12 nm) did not induce any toxicity after 14 days in SD-rats administered with a single oral dose (1959 or 2061 mg/kg bw) (Yun et al. 2015).

In the study of Li et al. (2015), M-SiNPs with different aspect ratios (AR, spherical 83 nm with AR 1, short rods with AR 1.75 and long rods with AR 5) were administered in mice (40 mg/kg bw via gavage). After 7 days, a very high amount of Si was detected only in the liver of spherical SiNPs exposed mice. Urinary excretion, intestinal absorption, and organ distribution of SiNPs were decreased with increasing aspect ratio. Furthermore, increase in renal damage such as hemorrhage, vascular congestion, and renal tubular necrosis with increasing aspect ratio was observed after 14 days of administration.

Kim et al. (2014b) exposed C57BL/6 mice (oral gavage; 750 mg/kg bw) to C-SiNPs (20 and 100 nm) that had been modified with or without l-arginine to determine the influence of surface charge on immunotoxicity in vivo. After 14 days, the author noticed a size-dependent decrease in WBCs cell count and cytokines (in blood), and reduced proliferation of B cells and T cells (from spleen) only for uncoated SiNPs.

#### Repeated exposure

C-SiNPs (12 nm) neither induce abnormal changes in blood biochemical and hematological parameters nor accumulated in any organs of the acutely (489.8, 979.5, or 1959 mg/kg bw/day, during 14 days) or sub-chronically (244.9, 489.8, or 975.9 mg/kg bw/day, during 13 weeks) exposed (via gavage) SD rats (Yun et al. 2015). In the study by Kim et al. (2014c), C-SiNPs (20 and 100 nm) did not induce any significant changes (compared to control) in clinical signs, blood biochemical, hematological, and histopathological analysis in sub-chronically exposed Crl: CD (SD) specific pathogen-free rats (500, 1000, or 2000 mg/kg bw/day for 90 days).

SD rats were administered (via oral gavage) with low doses (5, 10 or 20 mg/kgbw) of Pr-SiNPs (NM 200 and 201) and Py-SiNPs (NM 202 and 203) at 0, 24 and 45 h and sacrificed at 3 h after the last administration. No significant genotoxicity was observed in cells extracted from different organs (duodenum, colon, blood, kidney, liver, and spleen), and no pathological conditions were recorded in any of these organs (Tarantini et al. 2015a).

Hofmann et al. (2015) investigated the prenatal toxicity by exposing pregnant Wistar rats to Pr-SiNPs (NM 200) from gestation day 6–19. At doses of 100, 300, or 1000 mg/kgbw/day, administered SiNPs did not induce malformations in fetuses or death of the rats.

Zande et al. (2014) investigated the sub-acute and sub-chronic toxicity of Py-SiNPs (7 nm) and NM-202 (10–25 nm). In the sub-acute part, male SD rats were orally (via food) exposed to 100, 1000, or 2500 mg Py-SiNPs/kg bw/day for 28 days, while in the sub-chronic part, the rats were exposed only to the highest dose (2500 mg/kg bw/day) repeatedly for 84 days. ICP-MS analysis of target organs showed that NM-202 was significantly distributed in the lung, kidney, and spleen. Although no Si was detected in the liver, histopathological analysis and gene expression studies revealed that fibrosis was induced in the liver after 84 days of exposure. Si was found in the spleen of Py-SiNPs exposed rats only at the end of chronic exposure. The examined parameters such as blood biochemistry, antibody levels (IgG and IgM), and cytokines indicated no systemic toxicity in any of the Py- or NM-202 treated rats.

Yoshida et al. (2014) found an increased intestinal absorption of carboxyl (twofold) and amine-coated SiNPs (1.5-fold) compared to uncoated SiNPs (70 nm) in orally exposed BALB/c mice (2.5 mg/mouse/day for 28 days). In this study, the whole small intestine was processed using the everted gut sac method and a significant amount of Si was detected by ICP-MS. However, no signs of systemic toxicity were observed for any of these SiNPs.

In male Wistar rats that exposed to 10–15 nm SiNPs (oral gavage; 333.3 mg/kg bw/day for 5 days), histopathological analysis revealed gross tissue damage in kidney (cell swelling and necrosis), lung (interstitial pneumonia and bronchopneumonia), and in the testis (congestion, reduction of spermatogenesis and edema). In addition, blood biochemical parameters such as albumin, cholesterol, triglycerides, total proteins, urea, high-density lipoprotein (HDL), and low-density lipoprotein (LDL), as well as alkaline phosphatase (ALP) and aspartate aminotransferase (AST) activities were significantly increased in treated mice (Hassankhani et al. 2014).

### Inhalation exposure

#### Single exposure

In the study of Morris et al. (2016), C57BL/6 mice were intratracheally instilled with 4- or 20-mg S-SiNPs/kg bw and significant effects were observed only at the dose of 20 mg/kg. Twenty hours after instillation, approximately 20- and 10-fold higher cell number was observed in the bronchoalveolar lavage (BAL) of mice treated with the bare and amine-coated SiNPs, respectively, compared to control mice; neutrophils were also increased about 30- and 20-fold, respectively.

In another study, 15- and 55-nm C-SiNPs neither induced DNA damage nor micronuclei in lung and bone marrow (erythrocytes) cells of Wistar rats at 72 h of post-instillation (360 µg) (Maser et al. 2015). However, the distribution of administered SiNPs to these organs was not investigated.

#### Repeated exposure

A size- and dose-dependent increase in the distribution of SiNPs was observed in the serum and heart of male Wistar rats intratracheally instilled (2, 5, or 10 mg/kg bw/day for 16 days) with SiNPs (30, 60, 90, and 300 nm). Blood parameters (WBCs and platelets), inflammatory bio-markers (TNF-α, IL-1β, and IL-6), and oxidative stress bio-markers (ROS and MDA formation) were significant increased, while NO, NOS, and eNOS were significantly decreased in the serum of the treated mice (Du et al. 2013).

A study compared the toxicity and biodistribution of pristine SiNPs (19 nm) and aged paints containing SiNPs in BALB/c mice. The suspensions (20 µg/aspiration) were oropharyngeally aspirated once a week for 5 weeks, and mice were sacrificed either at 2- or at 28-day post-final aspiration treatment. Pristine SiNPs were significantly distributed in the lungs and liver, while only a low amount of Si was detected in the liver of paint-exposed mice. No signs of toxicity were observed, except a slight inflammation (a slight increase in macrophages and neutrophils together with an increase of IL-1β) with pristine SiNPs (Smulders et al. 2014).

Male Wistar rats were intranasally instilled with 150 µg SiNPs (10 and 80 nm) repeatedly for 30 days. After 30 days, a size-dependent increase in the levels of hydrogen peroxide (H_2_O_2_), MDA formation, TNF-α, IL-1β, MCP-1, and NF-ĸβ was observed in the frontal cortex, corpus striatum, and hippocampus of the brain. A similar quantity of Si was detected in all these three regions. In addition, a significant decrease in GSH levels was observed in these tissues (Parveen et al. 2015). The latter study shows the potential translocation of (mainly small) SiNP from the nose to the brain, but it has to be noted that the mice received a high dose during 30 days.

Intratracheally administered (2 mg/kg bw; 15 times, once every 3 days) S-SiNPs (58 nm) resulted in reproductive toxicity in C57 mice via enhanced ROS production and cell cycle arrest (G0/G1 phase) in the testicular tissues, and by decreasing the number of mature sperms and primary spermatocytes (Zhang et al. 2016). In another study, repeated intracheal instillation of S-SiNPs (58 nm; 0, 7, 21, and 35 mg/kg bw; 5 times, once every 3 days) induced local (macrophage activation in lung, liver, and spleen) and systemic inflammation (Increase in serum IL-8, TNF-α, and IL-6) in BALB/c mice. In addition, SiNPs were also detected in the lysosomes of macrophages in lung, liver, and heart tissues (Yang et al. 2016).

### Dermal exposure

#### Repeated exposure

The skin of SD rats was repeatedly exposed to different doses (500, 1000 mg, or 2000 mg/kg bw) of l-arginine coated C-SiNPs (20 nm) for 90 days (6-h exposure/day). The repeated exposure neither induced gross changes in the skin nor in any organs. In addition, hematological and blood biochemical parameters did not change in SiNPs-treated mice compared to controls (Ryu et al. 2014). In another study, Shim et al. (2014) showed that C-SINPS (20 and 100 nm) did not induce toxicity in the right and left brain or distribution in the cerebellum, hippocampus, or striatum of the dermally exposed rats (1000 or 2000 mg/kgbw daily for 90 days). In contrast to these studies, a significant increase in apoptosis (TUNEL positive cells) was observed in the skin of the BALB/c mice topically exposed to 70-nm SiNPs (250 mg/ear/day for 28 days). The author reported that the SiNPs were found not only in the skin but also in the regional lymph nodes, cerebral cortex, hippocampus, and in the liver (Nabeshi et al. 2011b).

### Parenteral exposure

#### Single exposure

In a 48-h study, exposure to 15-nm SiNPs (dose of 50 mg/kg bw by intravenous injection) resulted in a significant increase of CD68-positive Kupffer cells (KCs), WBCs, lymphocytes, monocytes, neutrophils, and TNF-α in the serum of male SD rats. There was a decrease in GSH activity and elevation in MDA levels in the liver of treated mice. Furthermore, bio-markers of liver dysfunction such as lactate, phosphorylcholine, sn-glycero-3-phosphocholine, tyrosine, phenylalanine, and lysine were increased in blood, while the levels of succinate, glucose, and glycine were significantly decreased in mice exposed to SiNPs (Chen et al. 2013).

In another study, 50 nm C-SiNPs (0.25 mg/kg bw) induced a significant increase in blood parameters such as leukocyte number, creatinine kinase (CK), ALT, AST, and LDH release in male tuck ordinary mice at 24-h post-intraperitoneal injection. Oxidative stress bio-markers such as MDA formation, SOD, and catalase were significantly increased in the lungs, liver, kidney, and brain of SiNPs-treated mice. Cytokines such as IL-6, IL-1β, and TNF-α were significantly increased in the lung and differentially expressed in other organs. Furthermore, all organs showed a significant DNA damage compared to saline treated mice and the extent of damage was in the order heart > kidney > lung > liver > brain (Nemmar et al. 2016).

In the study of Duan et al. (2014a, b), ICR mice were intravenously injected with 29.5, 103.5, or 177.5 mg/kg bw of 62 nm S-SiNPs. After 14 days, induction of autophagic vacuoles and mitochondrial rupturing in the heart tissues was observed using TEM. In addition, increase in LC3 positive staining in the heart tissues was detected at 103.5 and 177.5 mg/kg bw, and the expression of ICAM-1 and VCAM-1 was significantly decreased at 177.5 mg/kg. Huang et al. (2011) injected the mice intravenously with mesoporous rods (pure or PEGylated) at a dose of 20 mg/kg bw and found a dysfunction in biliary excretion and glomerular filtration. Blood analysis showed that there was a significant increase in total bilirubin (TBIL), blood urea nitrogen (BUN), and creatinine (CREA). The mesoporous rods mainly distributed in the lung, liver, and spleen, but PEGylation significantly reduced their distribution in these organs. In addition, short rods cleared via urine and feces more rapidly than long rods.

Yu et al. (2013) estimated the i.v. median lethal dose (LD_50_) for 64 nm C-SiNPs as 262 mg/kg bw in ICR mice using the Dixon’s Up and Down method. After 14-day post-administration, intravenously injected C-SiNPs (64 nm, 177.5 mg/kg bw) induced a significant increase in liver injury bio-markers (LDH, AST, and ALT). Histopathological analysis of target organs showed that the number of megakaryocytes in the spleen was significantly increased, and pulmonary hyperemia and interstitial thickening were observed in the lungs. Nearly 35% of the injected SiNPs were distributed in the spleen, 12.5% in the liver, and 2% in the lung of SNP-treated mice.

At 24 h of post-intravenous administration (30 mg/kg bw), TEM imaging of the BALB/c mice tissues showed that 70-nm SiNPs were localized in the regional lymph nodes, cerebral cortex, hippocampus, and the cytoplasm, and nucleus of hepatocytes (liver). Using in vivo imaging, the authors also found that the SiNPs were distributed near the liver immediately after the administration (20 min) and moved near the intestinal tract over time (6 h). Micron-sized silica particles (300 and 1000 nm) used in these studies were accumulated mainly around the gall bladder (Nabeshi et al. 2011b).

A significant increase in the levels of kidney injury bio-markers such as BUN and CREA and morphological changes associated with renal interstitial fibrosis was noticed in BALB/c mice intraperitoneally treated with 198-nm M-SiNPs (150, 300, or 600 mg/kg bw for 2 or 12 days). There was also a significant increase in the levels of fibronectin (FN), TGF-β and ICAM-1, and nuclear translocation of p65 in 300- or 600-mg/kg treated mice (Chen et al. 2015).

Zhuravskii et al. (2016) demonstrated that intravenously injected (70 mg/kg bw) Py-SiNPs (13 ± 5 nm) distributed and persisted in the liver of Wistar rats at 60-day post-administration. The authors also indicated that the administered SiNPs induced fibrosis and liver tissue remodeling by noticing the increase in blood ALT, and presence of mast cells, connective tissues, and foreign body-type granulomas in the liver.

#### Conclusion: in vivo

The in vivo toxicity studies have been carried out using rats and mice, and exposure through various routes of administration. In general, short-term exposure to SiNPs induced adverse effects in the lungs, kidneys, liver, and brain. SiNPs administered mainly via inhalation, ingestion, and intravenous routes were majorly distributed in the liver, lungs, spleen, and kidneys, and in the brain of the intranasally exposed rats. Most of the administered SiNPs were excreted via feces and to a lesser extent via urine, in a size- and shape-dependent manner. However, in most cases, the administered doses were very high compared to relevant human inhalation (Barsan 2007) and ingestion (Winkler et al. 2016; Dekkers et al. 2011) exposures to amorphous silica. Some studies showed accumulation of SiNPs in organs such as the liver, but such accumulation was not associated with any major effects. However, long-term effects of accumulated SiNPs were not studied. It is surprising that in contrast to acute studies, no toxicity (local or systemic) was observed in chronic oral and dermal exposure studies, regardless of the size of SiNPs and the high doses used. Moreover, surface-modified SiNPs showed a significant increase in absorption by the GI tract compared to bare SiNPs. Dosing SiNPs via i.v. showed in all studies some sign of damage/toxicity. No clear type-or size-dependent effect can be identified from the set of reviewed studies. Furthermore, where genotoxicity was studied, it was not clear whether SiNPs were taken up by the target cells or were able to reach the tissue examined, which is obviously needed to exert a direct genotoxic effect.

## Discussion

Growing production and use of SiNPs increase the risk of human exposures. Available toxicity studies mainly focused on effects after exposure via inhalation or skin (occupational exposure), ingestion (food additive), or nano-therapeutics (parenteral exposure). In vitro and in vivo studies demonstrated that SiNPs can induce adverse effects, but multiple ‘inconsistencies’ were found in the collected toxicity data set. As small differences in the physico-chemical properties of SiNPs could contribute to significant variation in the toxicity (Napierska et al. 2010), it is critical to discriminate how these variations influence toxicity. It is also worthy to note that the choice of cell type, culture system, assay conditions (Fede et al. 2012; Geys et al. 2010; Hayashi et al. 2017), and exposure route influence the toxic responses to SiNPs. However, the nature of these variations remains unclear, which continues to limit our understanding of SiNPs toxicity and hampers their hazard assessment.

### Physico-chemical characterization

In this review, a basic set of physico-chemical characteristics of primary SiNPs such as primary size, shape, crystallinity, and chemical composition (or) purity was set as inclusion criteria. Most studies associated the toxic endpoints to SiNPs size, while only a few related the toxicity to SiNPs porosity, shape, surface charge, and surface chemistry. Furthermore, hydrodynamic diameter is another widely reported property in these studies and the results suggest that, in most cases, SiNPs are often aggregated/agglomerated (AA) in cell culture media. Conversely, AA formation is also very likely in real-world matrices such as in air (Kim et al. 2014a), water, and in commercial products such as food (Dekkers et al. 2011). Such AA formation not only substantially alters the overall characteristics (such as size, shape and surface topology) but also potentially influences the biological outcomes (Luyts et al. 2013; Drescher et al. 2011). Although some progress has been made in the characterization of SiNPs AA in recent years (De Temmerman et al. 2012), their biological effects remain poorly understood.

### Toxicity of different types of SiNPs

Synthetic amorphous SiNPs are produced via different methods such as thermal (pyrogenic) or wet route (colloidal, precipitated and gel). The physico-chemical characteristics of SiNPs produced by these methods differ (Fruijtier-Pölloth 2012; Napierska et al. 2010) and may influence the biological outcomes. In this review, nearly 70% of the papers clearly reported the synthesis method. Among those 70%, about 80% reported the toxicity of wet method based SiNPs and only approximately 20% on the other types (such as pyrogenic and mesoporous). A general overview of toxic effects induced by different types of SiNPs is presented in Table 3. Cytotoxicity and genotoxicity induced by S- and C-SiNPs are strongly correlated with the induction of oxidative stress. For Pr-SiNPs, oxidative stress was associated with cytotoxicity but not genotoxicity. Interestingly, Py-SiNPs caused cytotoxicity, mostly without the generation of oxidative stress. In addition, recent studies showed that Py-SiNPs are biologically more reactive than C-SiNPs (Zhang et al. 2012) and Pr-SiNPs (Di Cristo et al. 2016) of same composition and size. It is known that C-, S-, and Pr-SiNPs are hydrophilic in nature, while Py-SiNPs are hydrophobic due to the de-hydroxylation of surface OH groups during the production process (Napierska et al. 2010), and such difference in surface chemistries might contribute to different biological activities. However, more systematic studies are required to verify these differences depending on production processes.

### Toxicity mechanisms of amorphous SiNPs and crystalline silica

A fundamental question was raised by Napierska et al. (2010): do amorphous SiNPs induce biological responses similar to crystalline silica? Oxidative DNA and membrane damage have been reported as the major toxic mechanisms involved in the health effects of micron-sized crystalline silica, which is also observed for C- and Pr-SiNPs. The latter two possess silanols on the surface while Py-SiNPs mostly contain siloxanes (Napierska et al. 2010). In addition, recent studies showed that the presence of surface moieties such as silanols is more correlated with crystalline silica toxicity than crystallinity (Zhang et al. 2012; Turci et al. 2016). Thus, silanols appear to be a common surface feature in C-SiNPs, Pr-SiNPs, and crystalline silica, which might contribute to a similar toxic activity. Furthermore, toxicity elicited by Py-SiNPs appears to be *oxidative stress*-*independent*, indicating that Py-SiNPs induce adverse effects via other mechanisms (Gehrke et al. 2013; Napierska et al. 2012a). More studies are required to verify these hypotheses.

### Influence of exposure routes in vivo

The exposure route obviously influences the in vivo absorption, biodistribution, and toxicity of SiNPs. For instance, after oropharyngeal aspiration or intratracheal instillation, the lungs are clearly the main target (Smulders et al. 2014), while after ingestion (Zande et al. 2014) or iv injection (Yu et al. 2013), the liver and/or the spleen were the targets. SiNPs accumulated in the brain of intranasally exposed rats (Parveen et al. 2015) but did not after oral exposure (Shim et al. 2014). In addition to the exposure route, physico-chemical properties such as size and shape clearly influence the clearance, distribution, and toxicity (Huang et al. 2011; Li et al. 2015).

### Adverse effects of chronic exposure in vivo

In repeated (oral and dermal) dose studies (≥28 days), SiNPs did not induce any local or systemic toxicity even in (very) highly dosed rats. A recent study demonstrated that mild and highly dosed rats excreted most of the orally administered SiNPs via the feces (Yun et al. 2015). In addition, Van der Zande et al. (2014) demonstrated the in vitro gelation of SiNPs with increasing concentrations, which might reduce the gastro-intestinal absorption in highly dosed animals and increase excretion via feces. Therefore, the use of (low) realistic exposure doses appears more appropriate, since the toxicokinetics may depend on the level of exposure (Paek et al. 2014). Moreover, information on the physico-chemical properties of ingested and digested SiNPs is lacking, representing a huge knowledge gap in the risk assessment of SiNPs in food (Dekkers et al. 2012).

### Correlation between in vitro and in vivo studies

Correlation between in vitro and in vivo effects is an indication that specific cells or tissues are potential targets for SiNPs toxicity. The results of in vitro and in vivo experiments (included within the same study) suggest that exposure to SiNPs could induce Kupffer cell mediated liver injury (Chen et al. 2013), kidney injury via the activation of NF-kB signaling pathways (Chen et al. 2015), and endothelial dysfunction via autophagy (Duan et al. 2014a). Several in vitro studies showed that SiNPs caused DNA double-strand breaks in a wide range of immortalized cell lines at low doses, but no such genotoxic effects were observed, even in animals at high doses. In addition, SiNPs did not induce micronuclei either in vitro or in vivo.

### Dosimetry in vitro

Accurate in vitro dosimetry is an important yet complex aspect of nanotoxicology (Lison et al. 2014). In many studies reviewed here, authors often tested high in vitro exposure doses. Considering the exposure dose as nominal dose may be appropriate only for well dispersed and stable SiNP suspensions (Lison et al. 2008). However, Py-SiNPs, for instance, often re-agglomerate in culture media with an effective density lower than the material density (Deloid et al. 2014), thereby potentially affecting the dose reaching the cells (Cohen et al. 2014). Thus, a realistic estimation of the delivered dose is necessary to compare the biological effects between studies and to establish good in vitro and in vivo correlations (Pal et al. 2015).

### Physico-chemical properties for the safer design of SiNPs

Immune responses after the administration of SiNPs are very crucial, as they can induce cascades of events by the secretion of cytokines, which may be harmful or beneficial. The data collected in this review show that the smaller the size, the stronger the pro-inflammatory effect. In addition to the size, the surface charge appears to play a role, since less negative charges seem to suppress the immune response. Porosity is another crucial factor influencing blood biocompatibility, i.e., the more porous the SiNPs, the less hemolysis of RBCs (Maurer-jones et al. 2010). Furthermore, SiNPs aspect ratio was shown to closely relate to in vivo organ retention and clearance (Huang et al. 2011). Therefore, the size, surface area, porosity, and geometry (shape) appear to be the key parameters for designing less-toxic and less-inflammagenic SiNPs, for bio-medical applications for instance.

In conclusion, SiNPs generally exhibit acute toxic effects in vitro and in vivo. The data on chronic effects of SiNPs exposure are rather conflicting with the acute effects and are still insufficient to draw firm conclusions. No concrete data were found to conclude whether amorphous SiNPs induce fibrosis like micrometric crystalline silica. Moreover, translation to human health effects is impossible at this moment due to the lack of realistic exposure and epidemiological data. Surface moieties (such as silanols, silanolates, and siloxanes) of SiNPs are found to be production process-specific and seem to be the key determinants of SiNPs toxicity. AA formation for some SiNPs is very dynamic in physiological media, but it is still unclear how it contributes to the hazard characterization. Therefore, the design of safe(r) SiNPs for food, medical, and other applications will only be possible when physico-chemical characteristics can be unambiguously linked to toxicity. Furthermore, detailed investigations on the SNP bioaccumulation/bioavailability and their long-term consequences in vivo are required for a safer use.
